# The allosteric mechanism leading to an open-groove lipid conductive state of the TMEM16F scramblase

**DOI:** 10.1038/s42003-022-03930-8

**Published:** 2022-09-19

**Authors:** George Khelashvili, Ekaterina Kots, Xiaolu Cheng, Michael V. Levine, Harel Weinstein

**Affiliations:** 1 Department of Physiology and Biophysics, Weill Cornell Medicine, New York, NY 10065 USA; 2 Institute for Computational Biomedicine, Weill Cornell Medicine, New York, NY 10065 USA

**Keywords:** Computational biophysics, Molecular modelling

## Abstract

TMEM16F is a Ca^2+^-activated phospholipid scramblase in the TMEM16 family of membrane proteins. Unlike other TMEM16s exhibiting a membrane-exposed hydrophilic groove that serves as a translocation pathway for lipids, the experimentally determined structures of TMEM16F shows the groove in a closed conformation even under conditions of maximal scramblase activity. It is currently unknown if/how TMEM16F groove can open for lipid scrambling. Here we describe the analysis of ~400 µs all-atom molecular dynamics (MD) simulations of the TMEM16F revealing an allosteric mechanism leading to an open-groove, lipid scrambling competent state of the protein. The groove opens into a continuous hydrophilic conduit that is highly similar in structure to that seen in other activated scramblases. The allosteric pathway connects this opening to an observed destabilization of the Ca^2+^ ion bound at the distal site near the dimer interface, to the dynamics of specific protein regions that produces the open-groove state to scramble phospholipids.

## Abstract (web summary)

Molecular dynamics simulations reveal the allosteric mechanism leading to an open, lipid scrambling competent state of a mammalian TMEM16F.

## Introduction

The TMEM16F belongs to the TMEM16 family of integral membrane proteins. The human genome encodes for 9 TMEM16 protein homologs that have been classified as Ca^2+^-activated phospholipid scramblases (PLS), or Cl^−^ channels (CaCC)^1–7^. The scramblase members of the family can also function as non-selective ion channels. They regulate the exposure of phosphatidylserine (PS) lipids on the cell surface and play essential roles in fundamental physiological processes, from blood coagulation, bone formation, and cell fusion to membrane repair and immune response^8^. Dysfunction of TMEM16 PLS proteins is associated with genetically inherited disorders of muscle^9,10^, bone^11–13^, blood^14,15^, and brain^16–19^, and mutations in TMEM16 PLS have been associated with various disease conditions^5,20–24^. Specifically, mutations in human TMEM16F (hTMEM16F) PLS are responsible for Scott syndrome^25^, a bleeding disorder caused by impairment of Ca^2+^-dependent externalization of PS lipids in activated platelets.

**Fig. 1:**
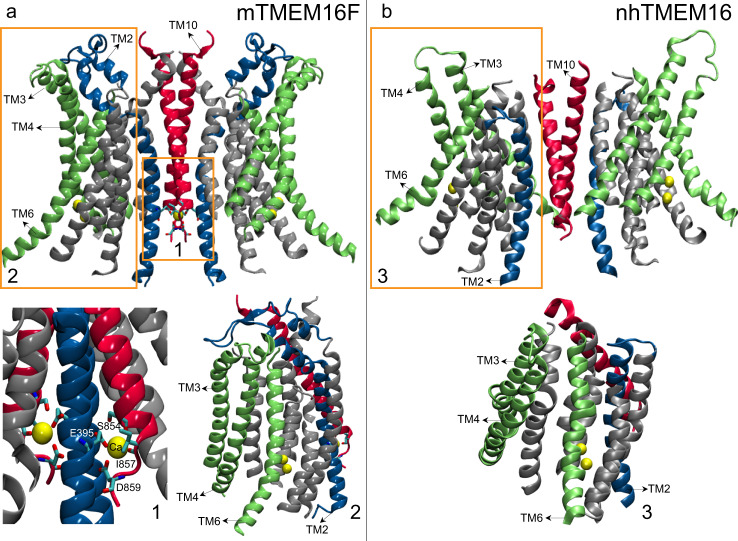
Distinguishing structural motifs of the TMEM16F PLS are highlighted by a comparison of mTMEM16F (PDBID 6QP6), in Panel a (*left*) and nhTMEM16 (PDBID 6QM9) in Panel b (*right*). Most of the intracellular and extracellular loop regions have been removed for clarity. In **a**, rectangle 1 highlights the third (distal) Ca^2+^ binding site at the dimer interface of mTMEM16F. Shown in licorice and labeled are the 4 residues coordinating the Ca^2+^ ion in its binding site (E395 on TM2, and S854, I857, and D859 on TM10). Rectangle 2 in **a** and Rectangle 3 in **b** highlight the groove regions in mTMEM16F and nhTMEM16, respectively. Shown are TMs 3, 4, and 6 (in green) lining the groove region, TM10 (in red), and TM2 (in blue). Note that the linker region connecting TMs 1 and 2 in the mTMEM16F model reaches towards the groove region and is positioned close to the extracellular end of TM4 helix (see also Fig. S1).

Much of what is known about structure/function relationships in TMEM16 PLS is inferred from breakthrough studies on fungal nhTMEM16 and afTMEM16 homologs^1,2,26–33^, but for mammalian TMEM16 PLS such structure-based functional insights are only now starting to emerge^6,34,35^. These insights have led to seemingly contradictory inferences, and it is still unclear to what extent the molecular mechanisms underlying activity or regulation of TMEM16 PLS are similar between mammalian and fungal homologs, or even among different mammalian ones. Thus, the X-ray and cryo-electronmicroscopy (cryo-EM) structures of Ca^2+^-bound fungal nhTMEM16 and afTMEM16 proteins^27,36^ have revealed a common homo-dimeric fold, in which the ten transmembrane helices (TMs) of each protomer generate a hydrophilic groove facing the membrane on the side opposite to the dimer interface (Fig. 1). This membrane-facing groove is lined by TMs 3–7 and connects (through TMs 6–7) to a pair of bound Ca^2+^ ions. In the absence of Ca^2+^ ions, the groove is occluded from the membrane as TM4 and TM6 helices reposition to close the groove. Structural, functional, and computational experiments on the fungal TMEM16 PLS^3,28,29,31–33,37,38^ revealed that in the presence of Ca^2+^ ions, the groove region in these scramblases can sample a wide range of conformations, such as an “ion-conducting” intermediate state (non-permissive to lipids but wide enough to conduct small size ions); a “membrane-exposed” open state (with an overall open conformation of the groove but still sufficiently narrow at the extracellular (EC) side to restrict the passage of lipid headgroups due to a constriction formed by the polar interaction network between TMs 3, 4, and 6); and a “lipid-conductive” state (in which the constriction is dissolved and the groove is transformed into a continuous hydrophilic conduit that allows the passage of lipids). Overall, these insights support the ‘credit card’ model mechanism for lipid scrambling^39^ by the TMEM16 PLS, whereby lipids traverse the bilayer by populating the hydrophilic groove pathway with their headgroups while keeping their hydrophobic tails perpendicular to the groove axis, in the bilayer environment.

More recent structure/function studies on mammalian hTMEM16K PLS^35^ have provided further support for this mechanism, as the Ca^2+^-bound groove in hTMEM16K was found to assume conformations wide enough to constitute a lipid translocation pathway. Indeed, MD simulations suggested that such an open-groove state in hTMEM16K is necessary for scramblase activity^35^. However, structural investigations of mouse TMEM16F (mTMEM16F)^6,34^ showed in striking contrast to the fungal TMEM16 PLS and the hTMEM16K, the groove in mTMEM16F remains closed even under conditions of maximal activation (i.e., Ca^2+^-bound and in the presence of highly anionic phosphatidylinositol 4,5-bisphosphate (PIP_2_) lipids). This led to the proposal that mTMEM16F PLS may function according to a different paradigm^34,40^, where lipid permeation occurs outside a closed scrambling pathway.

Comparison of existing structural data for the different TMEM16 PLS identifies two intriguing structural characteristics of mTMEM16F that together distinguish it from the fungal and hTMEM16K PLS (see Fig. 1 and Fig. S1): (1)-the closed groove in mTMEM16F on the EC side appears to be stabilized not only by TM4–TM6 interactions (referred here as TM4–TM6 interface as seen in the fungal and hTMEM16K PLS), but also by interactions between the EC end of TM4 helix and the extended helix-loop-helix motif on the EC end of TM2 (TM4–TM2 interface); and (2)-the mTMEM16F structure contains an additional, third Ca^2+^ ion bound at a “distal Ca^2+^ binding site” in the dimer interface region. The ion is stabilized at this site by the sidechains of two negatively charged residues—E395 on TM2, and D859 on TM10—as well as by two backbone carbonyls from residues S854 and I857 in TM10. While the analogous Ca^2+^ ion binding site exists in the hTMEM16K structures as well, the unique structural feature of mTMEM16F is that the intracellular (IC) end of TM2 coordinates the third Ca^2+^ ion while the EC end of the same helix participates in the interactions with TM4 to provide stability to the closed groove structure. These characteristics present an intriguing possibility that the distal Ca^2+^ ion site in mTMEM16F may be allosterically coupled to the EC side of the groove. Indeed, Ca^2+^ binding to the analogous site in the TMEM16A CaCC site, has been shown to facilitate TMEM16A channel opening^41^, suggesting the existence of an allosteric connection between the distal Ca^2+^ ion site and the groove region.

We, therefore, reasoned that dynamics of the Ca^2+^ ion in the distal binding site may allosterically affect conformational dynamics of the groove region in mTMEM16F and lead to its opening for lipid scrambling. To test this hypothesis we carried out massive, ~400 µs-long, all-atom ensemble molecular dynamics (MD) simulations of mTMEM16F. The analysis of the MD data revealed a gradual opening of the groove region and its transformation into a continuous lipid conduit. This conformational transition severs the interactions along the interfaces between TM4 and TM6, and between TM4 and TM2. Importantly, the analysis of MD trajectory data with Markov State Model (MSM) and transition path theory (TPT) approaches revealed that the unraveling of the TM4–TM2 interface was correlated in time with the destabilization of the Ca^2+^ ion in the distal binding site. To discern the mode of allosteric coupling between the ion binding site and the groove region we used N-body Information Theory (NbIT) analysis^42^ and quantified it with the thermodynamic coupling function (TCF) approach^43,44^. These revealed the allosteric path of communication between the two regions and showed that the mTMEM16F groove conformation that permits lipid scrambling is indeed energetically stabilized by the allosteric communication. These results provide specific mechanistic details in a structural context for the allosteric mechanism leading to an open, lipid conductive state of the groove in mTMEM16F.

## Results

### Opening of the mTMEM16F groove region is related to the restructuring of the distal Ca^2+^ binding site

A unique structural feature of mTMEM16F PLS, not observed in any available structural models of other TMEM16 scramblases, is the involvement of TM2 helix not only in coordination of the Ca^2+^ ion in its binding site at the dimer interface (distal Ca^2+^ binding site), but also in the apparent stabilization of the closed groove structure of mTMEM16F by interactions with TM4 (Fig. 1). Based on the span of this structural feature we hypothesized that it may support an allosteric connection between the intracellularly located, distal Ca^2+^ binding site, in mTMEM16F and the extracellular end of the groove. To test this hypothesis, we set out to probe whether the dynamics of the distal Ca^2+^ binding site allosterically affects conformational changes in the groove region to yield its opening. As described in “Methods”, the massive MD simulations totaling ~400 µs trajectory time were performed and analyzed using dimensionality reduction tICA formulation as described in “Methods”. The simulations were carried out according to a 3-stage adaptive protocol, in which each Stage is informed by the output from the previous one used to spawn swarms of multiple replicates (see “Methods”, Fig. S3). Each stage of the adaptive MD protocol was monitored for conformational changes in the distal Ca^2+^ ion binding site and the groove region. More specifically, the trajectories from Stage 1 simulations which migrated the farthest from the center of the population distribution of the tICA space were identified. From these, 48 trajectories were randomly selected for analysis (see Figs. S3 and S4). One of the monomers of mTME16F dimer in these frames was found to differ from the original structure in at least one of the following ways: (1) a destabilized Ca^2+^ ion in the distal site; (2) a widened TM2–TM4 interface; and (3) a widened TM4–TM6 interface. These frames were continued, in independent triplicates, in Stage 2. Following the same analysis protocol and criteria we selected 8 frames from the Stage 2 set for Stage 3 simulations, which also included a conformation from the highest population region (Figs. S3 and S4). In the course of this sequence of simulations, we observed a gradual opening of the groove region of one of the protomers of mTMEM16F that allowed lipid scrambling, concomitant with a destabilization of the Ca^2+^ ion in the distal binding site (Supplemental Movies 1 and 2).

#### Dimensionality reduction with tICA

**Fig. 2:**
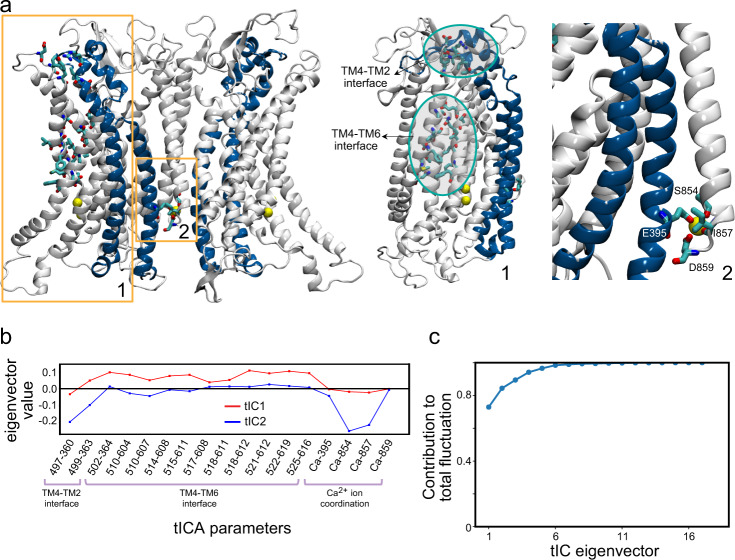
Description of the CVs used in the tICA dimensionality reduction analysis. **a** The three regions whose conformational changes are described with the chosen CVs are highlighted on the structure of the mTMEM16F model by the numbered rectangles: **1**. TM4-TM6 and TM4-TM2 interfaces of the groove; **2**. The distal Ca^2+^ binding site. In **1**, the specific residues whose pairwise distances throughout the trajectory served as CVs are shown rendered in licorice. In **2**, the four residues coordinating the Ca^2+^ ion (yellow sphere) in the binding site are shown rendered in licorice. **b** Contributions of the CVs used as tICA parameters to the tIC1 and tIC2 vectors. **c** Contribution of each tIC vector to the total representation of structural dynamics of the system.

**Fig. 3:**
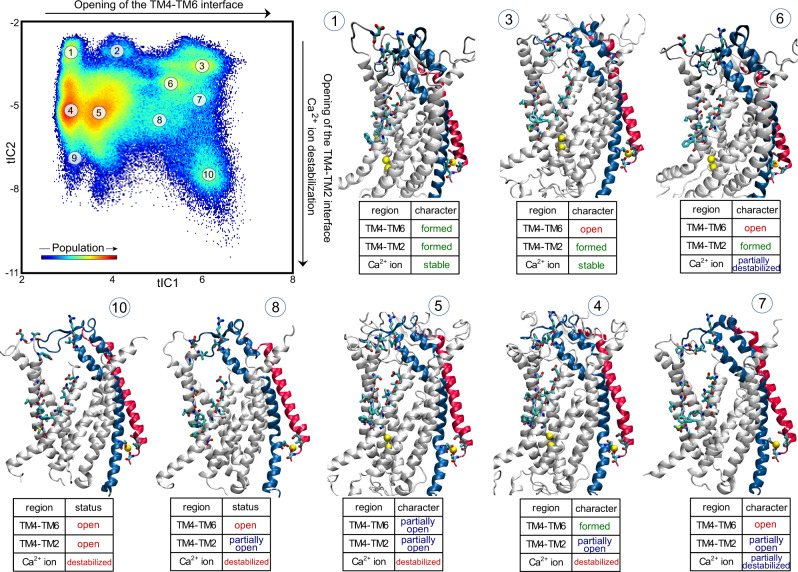
Structural interpretation of the tICA space features. Projection of all the MD trajectory frames from the adaptive ensemble simulations onto the 2D space spanned by the first two tIC vectors (see also Fig. S3). The color map identifies the populations distribution of the different states of mTMEM16F in this tICA space. Representative structures from selected microstates indicated on the tICA map are shown in the molecule models. The table accompanying each model describes the characteristics of the critical regions (TM4–TM6, TM4–TM2, and the distal Ca^2+^ ion binding site) in the corresponding microstate. Only one protomer of mTMEM16F is shown. TMs 2 and 10 are colored in blue and red, respectively. The Ca^2+^ ions are shown as yellow spheres and the relevant residues are drawn in licorice as in Fig. 2a. A more detailed structural quantification of the microstates is presented in Fig. S10.

To facilitate analysis of these conformational changes, we carried out tICA analysis by extracting from the MD trajectories three sets of dynamic CVs to quantify (i)-conformational changes at the TM4–TM6 and TM4–TM2 interfaces of the groove, and (ii)-changes in the coordination of the Ca^2+^ ion in its binding site (Fig. 2a, see “Methods”). The tICA transformation of the MD trajectory frames to the space of these variables showed that the first two tIC vectors (tIC1 and tIC2) represented >80% of the total dynamics in the system (Fig. 2c). Evaluation of contributions of each of the CVs to these vectors revealed that tIC1 encoded mainly structural changes at the TM4–TM6 interface, whereas tIC2 mostly encoded structural changes at the TM4–TM2 interface and in the distal Ca^2+^ ion coordination. Notably, these features of the tICA space were not affected upon probing with alternative choices of CVs, as illustrated in Fig. S8. Figure 3 presents the projection of all the MD trajectory frames onto a 2D space of the first two tIC vectors. Structural characteristics of the conformations sampled by the groove region and by the distal Ca^2+^ binding site in the simulations are indicated by the molecular representations of the selected microstates shown in the snapshots provided in Fig. 3. In Fig. S9 we show the results of discretizing this space into microstates as described in “Methods”, and results of the quantification of various collective variables in these microstates are given in the Fig. S10 histograms.

In the 2D tICA space spanned by the first two vectors, the opening of the TM4–TM6 interface is represented by microstates along the first eigenvector, tIC1 (Fig. 3 and S6). It can be divided into three parts: at small tIC1 values (microstates 1, 4, and 9), the TM4–TM6 interface of the groove is closed; at intermediate tIC1 values (microstates 2 and 5), it is partially open; and at large tIC1 values (microstates 3, 6, 7, 8, and 10), the TM4–TM6 interface is fully open.

Positions along the second eigenvector, tIC2, in Fig. 3 and S6 reflect the extent of opening of the TM4–TM2 interface, which can again be considered in three sections: as tIC2 values decrease from −2 to −11, the system first visits conformations with closed TM4–TM2 interface (microstates 1, 2, 3, and 6), which is followed by conformations with a partially open TM4–TM2 interface (microstates 4, 5, 7, and 8), and then by the fully open TM4–TM2 interface (microstates 9 and 10). This fully open structure of the groove contains a continuous membrane-exposed pore whose dimensions are comparable to those measured in the open groove structure of nhTMEM16^36^ (see Fig. S11).

A restructuring of the distal Ca^2+^ binding site is also occurring along tIC2. At values in the range from −2 to ~ −4 (microstates 1–3 in Fig. 3 and S6), the Ca^2+^ ion is stably bound in its site, coordinated by the sidechains of residues E395 and D859, as well as by backbone carbonyls of S854 and I857. But for tIC2 values <−4 (microstates 4–10), the Ca^2+^ ion is gradually destabilized as indicated by the loss of coordination with the backbone moieties of S854 and I857 (coordination with charged sidechains of E395 and D859 is maintained; see also Fig. S12a, b). Concomitantly, the Ca^2+^ ion becomes more hydrated (see “Ca^2+^ hydration” histograms in Fig. S10) and moves inward by ~5 Å while bringing with it the intracellular segment of TM2 (located below residue E395) and the terminal intracellular helical portion of TM10 (albeit to a lesser extent, see Fig. S12c). These conformational changes on the intracellular side are accompanied by the rearrangements of the extracellular end of TM2 which moves away from TM4 as the Ca^2+^ ion becomes destabilized. Thus, the tICA analysis records the evolution of the system from the ensemble of cryo-EM like states (microstate 1 in Fig. 3)—in which both TM4–TM6 and TM4–TM2 interfaces are closed and the distal Ca^2+^ binding site is intact—to the ensemble of conformations with both TM4–TM6 and TM4–TM2 interfaces of one of the monomers fully ruptured and the Ca^2+^ ion at the dimer interface destabilized and displaced from its binding site (microstate 10 in Fig. 3).

#### Analysis of kinetic pathways with MSM

**Fig. 4:**
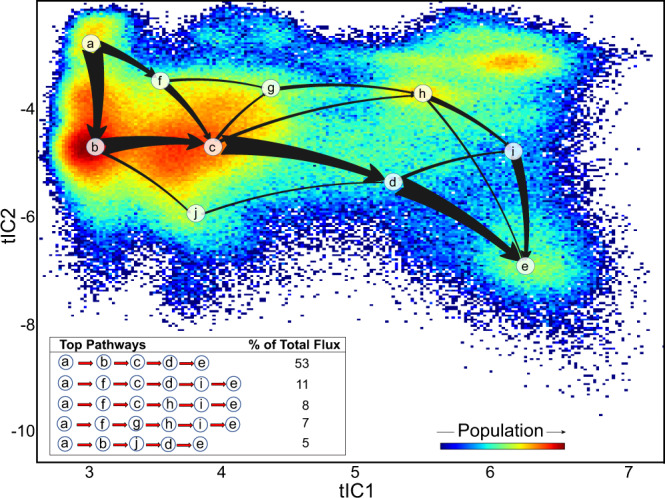
Kinetic pathways leading to groove opening states and destabilization of the distal Ca^2+^ ion at the dimer interface. The top 5 pathways identified from the TPT analysis to connect macrostate **a** (closed TM4–TM6 interface, closed TM4–TM2 interface, stably bound Ca^2+^ ion) to macrostate **e** (fully open TM4–TM6 and TM4–TM2 interfaces, destabilized Ca^2+^ ion) are identified in the table insert and represented by the arrows overlaid on the 2D space of the first two tIC vectors (see “Methods” and also Fig. S9 for macrostate assignment). The thickness of the arrows indicates the relative magnitude of the flux of the pathway. The relative contributions of the top pathways to the total flux values are given in the table.

To identify kinetic pathways leading to the groove opening and concomitant destabilization of the Ca^2+^ ion at the dimer interface, we built an MSM model on the microstates of the tICA space. As described in “Methods”, the microstates were lumped into 16 macrostates based on the MSM analysis (Fig. S9). The TPT analysis performed on these macrostates then identified top state transition pathways connecting structural states in the tICA space (see “Methods”), and revealed a dominant pathway leading to the structural changes described above (Fig. 4). Along this top pathway, the system first undergoes a transition from the ensemble of cryo-EM like states to the ensemble of conformations in which the TM4–TM6 interface of the groove is still intact, but the distal Ca^2+^ binding site is destabilized and the TM4–TM2 interface is partially open (in Fig. 4, see transition from macrostate **a** to **b**). This is then followed by the transitions to the states in which the TM4–TM6 interface begins to gradually open while the distal Ca^2+^ binding site remains destabilized and TM4–TM2 interface—partially open (in Fig. 4, see transitions from macrostate **b** to **c**, and **c** to **d**). Lastly, the system transitions to the ensemble of states in which both TM4–TM6 and TM4–TM2 interfaces of the groove are fully open and the distal Ca^2+^ binding site is destabilized (macrostate **d** to **e** transition in Fig. 4). The other top pathways identified from the TPT analysis largely follow a similar sequence of kinetic steps in which Ca^2+^ destabilization precedes full opening of the groove.

### The open groove conformations in mTMEM16F are stabilized by the allosteric coupling between the distal Ca^2+^ binding site and the groove region

As our results show that the opening of the groove in mTMEM16F is preceded by the restructuring of the distal Ca^2+^ binding site, we hypothesized that the groove opening is allosterically coupled to the dynamics of this site. To quantify such coupling, we first used the TCF formalism we developed^43,44^ (see “Methods”) to provide a quantitative description of how particular states, and transitions between them, are favored or opposed by allosteric coupling between reaction coordinates (i.e., CVs). As we had demonstrated that TCFs could be constructed in the context of MSMs, using the first tIC eigenvectors as CVs and the microstate free energies inferred from the MSM^43^, we used TCF here to quantify the allosteric coupling between the distal Ca^2+^ binding site and the groove opening.

**Fig. 5:**
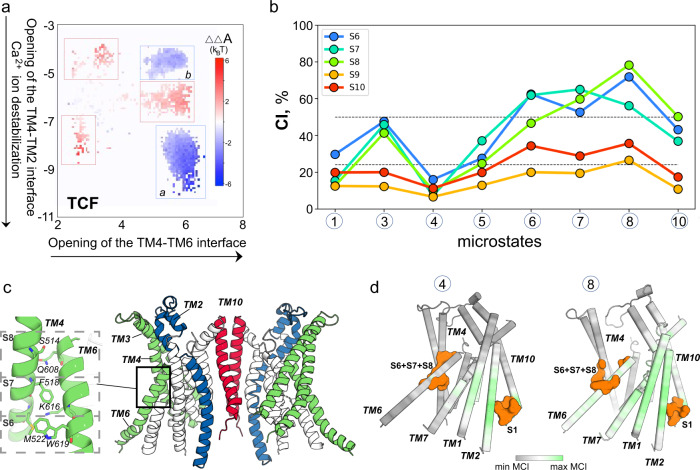
Allosteric coupling between the distal Ca^2+^ binding site and the groove region of mTMEM16F. **a** The 2D tICA space from Fig. 3 is shown colored according to the TCF. The regions that are stabilized by allosteric coupling between tIC1 and tIC2 vectors (∆∆*A*(*x, y*) < 0) are highlighted by blue rectangles and labeled as *a* and *b*, and the regions that are destabilized by such coupling ((∆∆*A*(*x, y*) > 0) are marked by red rectangles. **b** A plot of the CI values calculated between the *transmitter* S1 site (non-hydrogen atoms of the four Ca^2+^ coordinating residues: D395, S854, I857, D859) and several *receiver* sites at the TM4-TM6 interface (S6 site – non-hydrogen atoms of M522-W619 pair of residues; S7 site – non-hydrogen atoms of F518-K616 pair of residues; S8 site – non-hydrogen atoms of S514-Q608 pair of residues), calculated in the trajectories representing the indicated microstates (see also Fig. S13). The two horizontal lines demarcate regions of low, average, and high levels of coordination as obtained from the clustering of the *CI* data using the Fisher-Jenks algorithm (see “Methods”). **c** The mTMEM16F dimer structure highlighting the location and composition of the S6, S7, and S8 sites used in the calculations of *CI* shown in **b**. **d** The mTMEM16F monomer structure colored according to the normalized MCI values between the S1 site and the structural locus comprised of the residues from S6, S7, and S8 sites calculated for microstate 4 (left) and 8 (right) trajectories. The residue atoms at S1 and S6+S7+S8 sites are shown as orange surface.

Figure 5a shows the 2D tICA landscape colored according to the TCF values (∆∆*A*(*x, y*) calculated at each (*x*, *y*) grid point of the space (see “Methods”)). According to Eq. (1), the state (*x*, *y*) of the tICA space with ∆∆*A*(*x, y*) < 0 is stabilized by the thermodynamic coupling between tIC1 and tIC2 vectors, while the states with ∆∆*A*(*x, y*) > 0 are destabilized by such coupling^43^. The TCF map on Fig. 5a reveals two pronounced regions on the tICA space stabilized by the coupling between tIC1 and tIC2 (marked by blue rectangles *a* and *b*). The allosterically stabilized states include: (1) the states with fully open TM4–TM2 and TM4–TM6 interfaces (region *a*, the location of microstate 10 from Fig. 3), in which the Ca^2+^ ion is displaced from its binding site and coordinating contacts with S854 and I857 residues are lost, leading to a reorganization of the site (see Fig. S12); and (2) the state with fully open TM4–TM6 interface but closed TM4–TM2 interface and the Ca^2+^ stably coordinated by all four residues in the distal site (region *b*, the location of microstate 3 in Fig. 3). These two regions are separated, in the direction of tIC2, by a region of the space which is destabilized by the allosteric coupling between the two tIC vectors (red rectangle). This state corresponds to the conformations with fully open TM4–TM6 interface, destabilized distal Ca^2+^ binding site, but only partially open TM4–TM2 interface (location of microstate 7 in Fig. 3). Finally, two other states can be seen to be destabilized by the thermodynamic coupling between the tIC1 and tIC2 vectors: one corresponds to the conformations with closed TM4–TM6 interface but fully open TM4–TM2 interface and destabilized Ca^2+^ ion in the distal binding site (bottom left region of the tICA space marked by red rectangle), and the other corresponds to the conformations with the TM4–TM6 interface in the beginning stages of destabilization, but closed TM4–TM2 interface and intact distal Ca^2+^ binding site (top left region marked by red rectangle).

The two main mechanistic inferences from these results are: (i) under the conditions of destabilized Ca^2+^ ion in the distal site, the only conformations stabilized by the allosteric coupling between tIC1 and tIC2 are those with fully open groove (i.e., both TM4–TM6 and TM4–TM2 interfaces open); and (ii) the fully open TM4–TM6 interface can be allosterically stabilized under two conditions—the fully open TM4–TM2 interface and the reorganized distal Ca^2+^ binding site, or closed TM4–TM2 interface and intact distal Ca^2+^ binding site. Indeed, allosteric coupling between tIC1 and tIC2 makes it unfavorable for the fully open TM4–TM6 interface to exist under conditions of destabilized Ca^2+^ ion if the TM4–TM2 interface is only partially open.

### The dynamics of the distal Ca^2+^ binding site and the groove region are correlated during the groove opening process

To reveal the specific residue groups (structural motifs) involved in the pathway of allosteric coupling between the distal Ca^2+^ binding site and the TM4–TM2 and TM4–TM6 interfaces, we used the N-body Information Theory (NbIT) analysis formalism to calculate the coordination information (*CI*) between various sites (see “Methods”). The S1 *transmitter* site combined the residues coordinating the distal Ca^2+^ ion, while *receiver* sites in the S2–S10 sites defined in “Methods” for the TM4–TM2 (S2–S5 sites; Fig. S13) and TM4–TM6 (S6–S10 sites; Fig. 5c) regions contain residue pairs forming interfacial interactions in the closed groove conformation. These are disrupted during the conformational change (see Fig. S14 and “Methods”). The *CI* calculations used the trajectories representing the MSM metastable states identified to lie on the path from fully closed to opened conformations of the mTMEM16F groove (microstates 1, 4, 5, 6, 7, 8, and 10 in Fig. 3), as well as for the trajectory representing microstate 3 which was identified by the TCF analysis as one of the two regions of the tICA space stabilized by the allosteric coupling between tIC1 and tIC2 vectors (Fig. 5a).

#### Allosteric coupling to the TM4–TM6 interface

The *CI* values between the S1 site and the sites along TM4–TM6 interface (S6–S10) are given in Fig. 5b, while the *CI* values between the S1 site and the sites along TM4–TM2 interface (S2–S5) are shown in Fig. S13a. As can be seen from Fig. 5b, allosteric coordination between the S1 site and the individual sites along the TM4-TM6 interface (S6–S8) is relatively low for the states with a closed or partially open TM4–TM6 interface region (microstates 1, 4, and 5; see also Fig. S10), but it becomes high in the metastable states with a fully open TM4–TM6 interface (microstates 3, 6, 7, 8, and 10; Fig. S10). Especially notable are the high *CI* values in microstate 8 along the groove opening pathway. This microstate includes the ensemble of structures in which the TM4–TM6 interface is already open, the distal Ca^2+^ ion is fully destabilized, but the TM4–TM2 interface is still not fully open (Fig. S10). Interestingly, *CI* values between the S1 site, and sites S9 and S10 that combine residues from S6 to S8 sites located either on TM4 or on TM6 (Fig. 5c), are markedly lower in all the microstates (Fig. 5b). This suggests that the allosteric communication between the distal Ca^2+^ binding site and the TM4–TM6 region is not due simply to a translational motion of the TM4 and TM6 helices during the groove opening. Rather it is the result of changes in the sidechain fluctuations of the residues at the TM4–TM6 interface that are freed by the rupturing of TM4–TM6 interactions during groove opening.

#### Allosteric coupling to the TM4–TM2 interface

The analysis of *CI* between the S1 site and the S2–S4 sites along the TM4–TM2 interface (Fig. S13) reveals similar trends. Thus, while the coordination between these sites is overall weaker than that found for the S1 site and the sites along TM4–TM6 interface, we find that the *CI* values in Fig. S13 reach their peak again for the protein conformations representing microstate 8, highlighting the important role of this transition state for allosteric communication between the distal Ca^2+^ binding site and the groove region. Furthermore, *CI* between the S1 site and a site at the TM4–TM2 interface containing residues only from TM4 (S5 site, see Fig. S13), showed relatively weak coordination in all the microstates, suggesting that the allosteric communication between the distal Ca^2+^ binding site and the TM4–TM2 region is related to rupturing of the interactions along the TM4–TM2 interface during the groove opening process.

#### The allosteric pathways

To identify the channels of the allosteric communication identified between the distal Ca^2+^ binding site and the groove region, we quantified the *MCI* between the S1 site and the combination of residues in the S6, S7, and S8 sites along the TM4–TM6 interface (see “Methods”). As shown in Fig. 5d (right panel), *MCI* values calculated for the microstate 8 trajectory are high not only in the vicinity of the *transmitter* and *receiver* sites but also at other structural loci, most notably in helices TM1, TM2, TM6, and TM7, suggesting that the fluctuations throughout the protein are strongly coupled during the transition towards the groove opening. In contrast, the *MCI* values calculated from the trajectory of microstate 4 (in which the *CI* shows the Ca^2+^ binding site and the TM4–TM6 region to be weakly coupled to each other) are markedly lower (Fig. 5d, left panel). Together, the results from this NbIT analysis quantify—through specifically identified residue sets—the allosteric coordination of the groove region dynamics, to the dynamic changes in the distal Ca^2+^ binding site. The strength of this coordination is found to reach its peak in the metastable states on the tICA space that are visited on the groove opening pathway, connecting the findings from the tICA and NbIT analyses.

### The open groove conformation in mTMEM16F conducts lipids

**Fig. 6:**
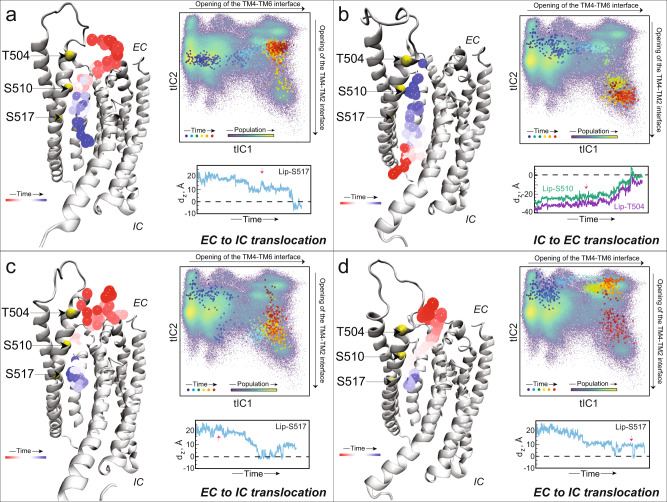
Events of lipid scrambling through the open mTMEM16F groove. The four panels depict lipid scrambling events from four different trajectories. **a**, **c**, and **d** Describe lipid translocation from the EC to IC side; **b** describes lipid translocation from the IC to EC side. In each panel, the protein monomer is the structure taken from the final frame of the respective simulation trajectory, shown in cartoon. The trajectory of the scrambled lipid is represented by the phosphorus atom shown as spheres colored according to the timestep (see “Time” color bar). The C_α_ atoms of residues T504, S510, and S517 are shown as yellow spheres. The time evolution of the corresponding MD trajectory projected onto the 2D tICA landscape from Fig. 3 is shown as large colored dots with darker colors (blue, cyan) indicating the initial stages of the simulation, lighter colors dots (yellow, green) corresponding to the middle part of the trajectory, and red shades showing the last third of the trajectory (the color map of the tICA space itself identifies the populations distribution of the different states of mTMEM16F with lighter and darker shades corresponding to the high and low density states, respectively). The directions on the tICA space along which TM4-TM6 and TM4–TM2 interfaces open are indicated outside the map, by arrows along the upper border and right border, respectively. The framed time plot in each panel shows the time-evolution of the distance between the phosphorus atom of the scrambled lipid as the Z-distance between the headgroup of the scrambled lipid and the C_α_ atom of residue S517 (d_Z_^S517^, blue trace) in **a**, **c**, and **d**. In **b**, the plot shows the distance of the headgroup and residues T504 and S510 (d_Z_^S504^ and d_Z_^S510^, purple and green traces, respectively). The lipid was considered scrambled in the EC to IC direction if during the trajectory d_Z_^S517^ became < 0, while in the IC to EC direction the lipid was considered scrambled if during the trajectory d_Z_^T504^ became > 0. The red arrows on the Z-distance plots mark the time-points in the trajectory when the TM4–TM2 interface of the groove is fully open (i.e., the system enters microstate 10). The headgroups of POPE and POPG lipid were defined as the center-of-mass of the following group of atoms (in CHARMM36 nomenclature): for POPE – N, C12, C11, P; for POPG – C13, OC3, C12, C11, and P.

The open, membrane-exposed conformations sampled by the mTMEM16F groove in the MD simulations are similar to experimentally obtained open groove structures in the other TMEM16 scramblases (e.g., in nhTMEM16, see Fig. S11), suggesting that the observed open groove state in mTMEM16F would support lipid scrambling. Quantification of the overall number of lipid phosphate headgroups inside the groove volume showed that, in general, the open groove in mTMEM16F can accommodate as many as 4 lipid headgroups at the same time (in Fig. S10, see “Lipid Count” histograms for the microstates located at tIC1 values >5). This number consistent with that reported from MD simulations of nhTMEM16^28,29,31,32^. Importantly, we observed as well multiple scrambling events through the mTMEM16F open groove structure. Figure 6 details the steps of the lipid scrambling process in four separate MD trajectories (see the figure captions for the criteria used to classify a lipid translocation through the groove as a full scrambling event; also note that due to velocity resampling between the stages, the trajectories are continuous in the position space, but discontinuous in the momentum space). In 3/4 cases, the lipid scrambled through the open groove in the EC to IC direction (panels a, c, and d, Fig. 6), while in the fourth trajectory it scrambled in the opposite direction, i.e., IC to EC (panel b, Fig. 6). As detailed in Fig. S10 by the histograms of distances at the TM4–TM6 and TM4–TM2 interfaces, all 4 of the trajectories projected onto the 2D tICA space in Fig. 6 visit the regions characterized by large distances at these open interfaces.

As expected from the dynamic properties of the system described above, the initial stages of the IC to EC scrambling process (Fig. 6b) are associated with a gradual widening of the TM4–TM6 interface and the partial opening of the TM4–TM2 interface; at this point in the trajectory, the translocated lipid is still in the IC part of the groove. Midway through the trajectory, both the TM4–TM6 and the TM4–TM2 interfaces open fully (see red arrow on the *d*_*Z*_ plot in Fig. 6b) as the system enters microstate 10. Concomitantly, the lipid moves towards the EC vestibule to complete the flipping process (i.e., its headgroup reaches the level of the C_α_ atom of residue T504 located at the very tip of the TM4 helix; see time-evolution of *d*_*Z*_ plots). The opening of the TM4–TM2 interface appears to facilitate this last step of the scrambling process. Indeed, in a different trajectory, in which the TM4–TM6 interface fully opened but TM4–TM2 remained closed, a lipid traveling from the IC to EC side performed only a partial flip. This is shown in Fig. S15 where the translocated lipid reaches the EC side of the groove and geometrically flips (Fig. S15d, e) but remains in the groove at >10 Å below the level of the T504 (Fig. S15c).

In the three trajectories in which lipid scrambling in the EC to IC direction was observed (Fig. 5a, c and d), the opening of the TM4–TM2 interface was seen to facilitate partitioning of a bulk lipid from the extracellular leaflet into the groove area to start the translocation process downward towards the IC end. In all three trajectories the TM4–TM2 interface opens partially in the early stage of the simulations, as can be inferred from the projections of the trajectories onto the 2D tICA space (locations of dark-colored dots, see also Fig. S10). This allows a lipid diffusing near the protein to partition into the EC side of the groove by engaging with positively charged residue K370 on the EC end of TM2 (Fig. S16, time-plot “Lip-K370”, and the snapshots at timepoints a, b).

In two of the three trajectories (Fig. 6a, c), these events are followed by the full opening of both TM4–TM2 and TM4–TM6 interfaces (the systems sample microstate 10). From this point in the trajectory onward, the lipid from the EC side starts traveling down the groove to flip (see the red arrow in *d*_*Z*_ plots in Fig. 6a, c). In the third trajectory (Fig. 6d), the lipid that entered the EC side of the groove in the early stages of the trajectory moves gradually down the groove and completes the flip after the TM4–TM2 interface opens fully (see the red arrow in *d*_*Z*_ plot in Fig. 6d). In all the trajectories, as the translocated lipid travels through the groove, it is coordinated with another positively charged residue, K616 on TM6 (Fig. S16, time-plot “Lip-K616”, and the snapshot at timepoint c).

Together, these results provide detailed mechanistic descriptions under the simulation conditions, of the key steps in the scrambling process of lipids through the open groove conformation of mTMEM16F that forms spontaneously through an allosteric process.

### The groove of mTMEM16F with Ca^2+^ ions bound in the primary site can open fully in the absence of Ca^2+^ bound in the distal site

Because our results show that destabilization of the Ca^2+^ ion in the distal site has a mechanistic role in mTMEM16F groove opening when the primary Ca^2+^ site is occupied, we investigated whether the groove can fully open when the distal Ca^2+^ site has lost its bound ion. To this end, we performed MD simulations of the mTMEM16F lacking the Ca^2+^ ions in the distal sites. The starting points for these simulations were two separate frames from the original simulation set (see Fig. S17) in which the groove is partially, but not yet fully, open (as can be judged by the location of the projections of the frames on the tICA surface in Fig. S17). The 30 independent replicates generated from each frame (with randomly assigned velocities), were run for 616 ns each, for a cumulative MD time of ~37 μs. As can be seen from the phase space sampling in Fig. S17, the Ca^2+^-free system still explores the region with the fully open groove (lower right part of the tICA space, the location of microstate 10; see Fig. 3). Furthermore, as shown in the structural snapshots in Fig. S17, the groove opening involves the same structural rearrangement of the intracellular end of TM2 helix as observed under conditions of Ca^2+^-bound distal site, shown in Fig. S12 (in Fig. S17 compare gray and yellow structures—RMSD over all the TM helices is 1.1 Å).

Since these results suggest that the groove opening mechanism is functional when the distal binding site is empty, we asked if an mTMEM16F system with a destabilized Ca^2+^ ion in the distal site can lose it as the process unfolds. To address this question, we ran ensemble MD simulations of 60 structures identified on the tICA space (Fig. 3) to represent centers of the microstates. Each of these models (6 replicas of a center of each of the 10 microstates) was run for ~430 ns (total sampling time of ~26 μs). Figure S18 shows results for the trajectory in which Ca^2+^ unbinding from the distal site was observed. Although Ca^2+^ unbinding occurred rarely in this set of simulations (once in 60 replicates), our findings represented in Figs. S17 and S18 indicate that the unbinding of this Ca^2+^ ion may facilitate the transition to the open groove conformation.

## Discussion

The results of the computational experiments described here demonstrate that the closed groove structure in mTMEM16F can spontaneously transition into an open, membrane-exposed conformation that supports lipid scrambling. While the properties of open and closed groove conformations in TMEM16F have been previously explored with simulations^45^, those studies employed structural models built by homology to the known open and closed groove structures of the fungal nhTMEM16 PLS. In contrast, we report here in atomistic detail the dynamic changes that lead from the closed groove structure of mTMEM16F to a scrambling-competent open groove state of this system in MD trajectories totaling ~400 µs, which allow us to define and quantify the allosteric mechanisms and kinetic model of the process. Thus, we show that the groove opening in mTMEM16F is mechanistically enabled by the allosteric coupling between the two structural motifs that are uniquely present in mTMEM16F but have not been observed together thus far in any available experimentally determined structures of other TMEM16 PLS. One of these motifs is the distal Ca^2+^ ion binding site at the IC end of the dimer interface. The coordination shell of the Ca^2+^ ion at this location includes two acidic residues of which one, E395, resides on TM2. The second structural element involves the extended helix-loop-helix motif which connects the same TM2 helix to TM1 at its other, EC, side. This linker region in mTMEM16F is much longer than the analogous loop region in fungal TMEM16 PLS or in hTMEM16K and reaches over the groove area to contact the EC end of TM4 (Fig. S1). This interaction appears to provide additional stability to the closed groove structure in mTMEM16 which is mainly stabilized by contacts between the middle regions of TM4 and TM6 helices.

Remarkably, we find that this situation changes upon the destabilization of the Ca^2+^ ion in its binding site observed in the MD trajectory, which allosterically triggers the disengagement of TM4 from TM2 at their interface. Our analysis showed these two processes to proceed in temporal accord: as the Ca^2+^ ion is displaced from its binding site and moves towards the protein interior, so does the IC part of TM2 as residue D359 maintains the ion coordination. Concomitantly, the EC end of TM2 helix moves away from TM4. These processes are followed by the gradual widening of the TM4–TM6 interface and full disengagement of TM4 from TM2. In this sequence of conformational transitions, the mTMEM16F groove becomes a continuous, membrane-exposed hydrophilic conduit which can be populated, and traversed, by lipid headgroups.

The opening of the TM4–TM2 interface appears to be facilitating the scrambling process from the EC region of the groove in an initial step of groove enlargement that is reminiscent of the mechanistic role of the polar interaction network on the EC ends of TM4 and TM6 helices in nhTMEM16. As we concluded from our MD simulations of the fungal PLS^29^, the network of polar residue interactions at the EC end of the nhTMEM16 groove, forms a constriction that blocks the passage of lipid headgroups. We showed that dissolution of this interaction network was necessary to trigger full groove opening for lipid translocation through the nhTMEM16 groove^29^. As mTMEM16F PLS does not contain such a network of interactions, we propose that a similar mechanistic role can be played here by the interactions between the EC parts of TM4 and TM2. Because the opening of the TM4–TM2 interface is seen to be allosterically connected, through the TM2 helix, to the dynamic rearrangements in the distal Ca^2+^ binding site, we suggest that in mTMEM16F the open/close groove equilibrium is regulated by the dynamics of the distal Ca^2+^ ion. Specifically, when the distal site is engaged with Ca^2+^, the groove is likely to be in the closed conformation and therefore scrambling activity will be minimal. Upon destabilization/unbinding of this Ca^2+^ ion, and in the presence of Ca^2+^ bound in the canonical site, the transition to the open groove conformation becomes energetically more feasible under the simulation conditions.

It should be intriguing to establish the existence of such a regulatory mechanism in other, as yet structurally uncharacterized, TMEM16 scramblases that may contain the two key structural features described above. A possible candidate is TMEM16E, which is highly homologous to TMEM16F (hTMEM16E bears ~50% sequence identify to mTMEM16F). It is clear, however, that the same type of allosteric communication is unlikely in the fungal TMEM16 PLS, which lack a distal Ca^2+^ ion site, or in hTMEM16K^35^, in which TM2 does not directly engage in groove stabilization and thus is not expected to be on an allosteric path from the distal Ca^2+^ ion to groove opening.

Because recent studies of the TMEM16A Cl^-^ channel member of the family have shown that Ca^2+^ ion binding at an analogous distal site in this protein was able to facilitate allosterically the channel opening process^41^, we note that the Ca^2+^ ion coordination shell in TMEM16A consists of three anionic residues in contrast to the coordination shell in mTMEM16F which contains only two anionic sidechains (in TMEM16A, the residue in the position analogous to S854 in mTMEM16F is replaced by Asp). The additional negative charge may entail a different mode of interaction with the protein and Ca^2+^ stabilization. It will be of interest to compare the allosteric communication between the distal Ca^2+^ binding site and the groove region to the findings presented here.

Thus far, our studies of TMEM16F functional dynamics have not addressed the role of specific lipids, although earlier work had suggested involvement of PIP_2_ lipids in TMEM16F scramblase activity^34^, and a regulation of scrambling mechanisms of TMEM16 PLS by their lipid environment has been demonstrated^26,27,40^. The possibility that lipids with different tail lengths and saturation may affect the scrambling by an open-groove state of TMEM16F remains open, as is that of an alternative mechanism of lipid scrambling that envisions lipid translocation with a closed groove^34^. The open groove scrambling mechanism uncovered in our work may complement the proposed closed groove scrambling mechanism. An interplay between these mechanisms in the context of different lipid environments remains a tantalizing possibility yet to be explored.

## Methods

### Molecular constructs for molecular dynamics (MD) simulations

The all-atom MD simulations described in this work are based on the cryo-EM structure of the mTMEM16F protein (PDBID: 6QP6^6^) in which each monomer includes two Ca^2+^ ions bound in the primary binding site, and a third Ca^2+^ ion bound in the distal site. The missing segments from the structure (1–42, 150–186, 489–502, and 876–911) were modeled using Rosetta’s AbinitioRelax application^46^. For the 876–911 segment, 1000 structures were generated and clustered. The most populated cluster (with clustering threshold of 4 Å) consisted of 315 structures, in all of which the 891–904 fragment was alpha helical. Based on the positioning of the analogous helical fragment in the structure of fungal homolog nhTMEM16 (PDBID 6QM5^47^), we positioned the 891–904 segment of mTMEM16F to interact with the N-terminal part of the opposite monomer (near residues 30–39, see Fig. S2) in the starting structure of the simulations. The 428–444 missing stretch in the 6QP6 structure was modeled as a hairpin connecting TM2 and TM3 using Modeler v9^48^ with nhTMEM16 (6QM5)^47^ and hTMEM16K(5OC9)^35^ as structural templates. In the completed structure, the first 20 N-terminal 20 were excluded due to their high flexibility, so that the final model contained residues 21–911 (Fig. S2). Protonation states of the titratable residues were predicted with Propka 3.1^49^ at pH 7, which resulted in histidines 275, 655, and 818 being modeled as HSE type, while the other histidine residues were modeled as HSD type. According to the annotation given in the PDBID:6QP6, disulfide bonds were introduced between the following pairs of Cys residues: 331–372, 338–365, 349–807, and 596–601.

Using the CHARMM-GUI web interface^50^, the mTMEM16F model was embedded into a lipid membrane consisting of a 7:3 mixture of POPE (1-palmitoyl-2-oleoyl-sn-glycero-3-phosphoethanolamine) and POPG (1-palmitoyl-2-oleoyl-sn-glycero-3-phospho-(1′-rac-glycerol)) lipids, the same lipid composition as used in our previous simulations of nhTMEM16 PLS^26,28,29^. The protein to lipid ratio was 1:1050. After adding a solvation box containing 150 mM K^+^Cl^−^ the total system contained ~ 561,219 atoms.

### Atomistic MD simulations

The assembled system was subjected to a short equilibration run with NAMD 2.13^51^ using a standard set of equilibration scripts provided by CHARMM-GUI. After this initial equilibration, the velocities of all the atoms were randomly regenerated and the system was subjected to an extensive 3-stage adaptive ensemble MD simulation protocol (Fig. S3). In Stage 1, the system was simulated in 300 independent replicates, each ~200 ns long. The analysis of the trajectories to assess the extent of conformational sampling (see “Results”) identified 48 frames for the next round of simulations (Fig. S4a). In Stage 2, these 48 structures were run in three independent replicates (144 replicates in total), each for ~1.5 µs. Another round of analysis further identified 8 trajectory frames from Stage 2 simulations to be considered for the next iteration (Fig. S4b). Thus, in Stage 3 of ensemble simulations, these 8 frames were run in 18 independent replicates (144 replicates in total), each for ~800 ns. The velocities of all the atoms were resampled from a Maxwell-Boltzmann distribution at the start of each stage of the protocol. Overall, this multi-stage adaptive protocol resulted in a net sampling time of ~400 µs.

The ensemble MD simulations were carried out with OpenMM 7.4^52^ and implemented PME for electrostatic interactions. The runs were performed at 310 K temperature, under NPT ensemble using semi-isotropic pressure coupling, and with 4 fs integration time-step (with mass repartitioning). Monte Carlo barostat and Langevin thermostat were used to maintain constant pressure and temperature, respectively. Additional parameters for these runs included: “friction” set to 1.0/picosecond, “EwaldErrorTolerance” 0.0005, “rigidwater” True, and “ConstraintTolerance” 0.000001. For the van der Waals interactions we applied a cutoff distance of 12 Å, switching the potential from 10 Å.

For all simulations, we used the latest CHARMM36m force-field for proteins and lipids^53^, as well as the recently revised CHARMM36 force-field for ions which includes non-bonded fix (NBFIX) parameters^54^.

### Dimensionality reduction with the tICA approach

To facilitate analysis of conformational dynamics in the simulations, we performed dimensionality reduction using tICA (time-lagged independent component analysis)^55^ as previously described^28,29,56,57^. Briefly, in the tICA approach the MD simulation trajectories are used to construct two covariance matrices: a time-lagged covariance matrix (TLCM) *CTL(τ)* = *<X(t)XT(t* + *τ)>*, and the usual covariance matrix *C* = *<X(t)XT(t)>*, where *X(t)* is the data vector at time *t*, *τ* is the lag-time of the TLCM, and the symbol *<…>* denotes the time average. To identify the slowest reaction coordinates of the system, the following generalized eigenvalue problem is solved: *CTLV* = *CVΛ*, where *Λ* and *V* are the eigenvalue and eigenvector matrices, respectively. The eigenvectors corresponding to the largest eigenvalues define the slowest reaction coordinates. These reaction coordinates depend on the choice of data vector *X*, i.e., the choice of collective variables (CV). Here, to define the tICA space, we considered CVs that quantify conformational dynamics of the TM4–TM6 and TM4–TM2 interfaces of the mTMEM16F groove, as well as the coordination of the Ca^2+^ ion bound at the third binding site, referred throughout simply as the third Ca^2+^ ion (see “Results”, Fig. 1). Thus, the following 3 sets of dynamic measures were extracted from the analysis of the MD trajectories to serve as CVs: (1) the C_α_–C_α_ distance between residue pairs 502–364, 510–604, 510–607, 514–608, 515–611, 517–608, 518–611, 518–612, 521–612, 522–619, and 525–616 (TM4–TM6 interface); (2) the C_α_–C_α_ distance between the residue pairs 497–360, and 499–363 (TM4–TM2 interface); (3) any minimum distance between the third Ca^2+^ ion and residues E395, S854, I857, and D859.

### Markov State Model (MSM) construction

To perform MSM analysis, we used Python 2.7.14 scripts included in MSMBuilder software^58,59^. Briefly, the 2D tICA space was discretized into 50 microstates using automated clustering *k-means* algorithm, and a transition probability matrix (TPM) was built^58^. To ensure Markovian behavior, multiple TPMs were constructed for different time intervals between transitions (MSM lag times), and the relaxation timescales of the system were calculated as ${\tau }_{i}=\tau {\prime} /{{{{{\rm{ln}}}}}}{\lambda }_{i}$, where *τ*′ is the lag-time used for building the TPM, *λ*_*i*_ denotes the *i*th eigenvalue of the TPM, and *τ*_*i*_ represents relaxation timescale (implied timescale) corresponding to the *i*th relaxation mode of the system. The Markovian property of the TPM was established by verifying the independence of *τ*_*i*_ from *τ*′. This analysis, presented in Fig. S5a, identified 160 ns as the lag-time at which the implied timescales begin to converge. To test whether a 160 ns lag-time was indeed appropriate, we performed the Chapman–Kolmogorov test using PyEMMA software^60^. As shown in Fig. S6, using *τ*′ = 160 ns lag-time, the model predictions match well with the observed dynamics. Further, we compared the top MSM relaxation modes for lag times of 160, 320, and 480 ns. As illustrated in Fig. S5b, the similarly ranked relaxation modes for the different lag-times represent the same dynamics of the system (i.e., the exchange of populations occurs between the same set of microstates regardless of the lag-time). Lastly, we compared the equilibrium population distributions predicted by the MSM built with 160 ns lag-time with the population distributions from the tICA space. The results shown in Fig. S7 reveal good agreement between the two populations. Thus, we concluded that *τ*′ = 160 ns is a valid lag-time for the MSM analysis.

### Transition path theory (TPT) analysis

In order to identify the most probable pathways, we applied TPT analysis as previously described^57^. Briefly, using a Robust Perron Cluster Analysis (PCCA+) algorithm^61^, the 50 microstates within the tICA space were clustered into 16 macrostates based on their kinetic similarity. Using the Dijkstra graph theory algorithm^62^ implemented in the MSMbuilder software, a flux matrix^63^ was then constructed for macrostates, and the most probable pathways were identified as those with the highest flux between the starting macrostate and a final microstate.

### Thermodynamic coupling function (TCF) analysis

The TCF analysis was carried out as previously described^43,44^. Briefly, given two CVs, $X(\overrightarrow{r})$ and $Y(\overrightarrow{r})$, which are functions of only the atomic coordinates, $\overrightarrow{r}$, the TCF identified as ∆∆*A*(*x, y*), is a function of specific values (*x, y*) of those CVs:

1 $$\varDelta \varDelta A(x,y)=-{k}_{{{{{\rm{B}}}}}}T{{{{{\rm{log}}}}}}\,\left(\frac{p(x,y)}{p(x)p(y)}\right)$$

In the above, *k*_B_ is the Boltzmann constant, *T* is the temperature, and *p(x,y), p(x),* and *p(y)* are, respectively, the observed joint and marginal probability distributions of molecular conformations. Here, $X(\overrightarrow{r})$ and $Y(\overrightarrow{r})$ were calculated from the normalization of the first two tIC vectors as^43^:

2 $$X(\overrightarrow{r})=\frac{tIC1\,(\overrightarrow{r})}{{{{{{\rm{ceiling}}}}}}({{{{{\rm{max}}}}}}(tIC1))-{{{{{\rm{floor}}}}}}({{{{{\rm{min}}}}}}(tIC1))}$$

3 $$Y(\overrightarrow{r})=\frac{tIC2\,(\overrightarrow{r})}{{{{{{\rm{ceiling}}}}}}({{{{{\rm{max}}}}}}(tIC2))-{{{{{\rm{floor}}}}}}({{{{{\rm{min}}}}}}(tIC2))}$$

Each configuration in microstate *m* was given a weight:

4 $$w(m)=\frac{p(m)}{{N}_{m}}$$

where *p*(*m*) represents the equilibrium weight of the microstate taken from the MSM, and *N*_*m*_ is the number of configurations associated with this microstate. The 2D space of the first two tIC vectors was then divided into 100 equally spaced bins in each direction, and the joint probability density corresponding to a bin centered at (*x, y*) was estimated over the configuration probability density within that bin^43^:

5 $$p(x,y)\approx {\sum }_{i=1}^{N}w({\Omega }_{m}({\overrightarrow{r}}_{i}))$$

where ${\Omega }_{m}({\overrightarrow{r}}_{i})$ is the index of the microstate a given configuration resides in, and *f*$({\overrightarrow{r}}_{i})=\,w({\Omega }_{m}({\overrightarrow{r}}_{i}))$ represents the probability density function over the configurations $\overrightarrow{r}$. From Eq. (5) above, *p*(*x*) and *p*(*y*) were calculated by summing over the corresponding dimensions.

Estimation of confidence levels for each bin was performed using the bootstrapping procedure described previously^43^.

### N-body information theory (NbIT) analysis

To quantify the allosteric coupling between the distal Ca^2+^ binding site and the mTMEM16F groove region we have applied N-body Information Theory (NbIT) analysis^42^ to the trajectories of metastable MSM microstates which represent local energy minimum conformations on the tICA space (see “Results”). The NbIT method relies on the Information Theory representation of configurational entropy *H* calculated from the covariance matrix (***C***) of all atomic positions (**X**) in the protein:

6 $$H(X)=\frac{1}{2}\,{{{{\mathrm{ln}}}}}|2\pi e{{{{{\boldsymbol{C}}}}}}({{{{{\boldsymbol{X}}}}}})|$$

Total correlation (*TC*), defined as the total amount of information that is shared among *N* atoms in a set, was then quantified as:

7 $$TC({X}_{1},\ldots ,{X}_{N})={\sum }_{i=1}^{N}H({X}_{i})-H({X}_{1},\ldots ,{X}_{N})$$

where *X*_*i*_-s represent components of 3N-dimensional vector corresponding to *x*, *y*, *z* atomistic coordinates of the atoms in the set, and *H*(*X*_1_,…,*X*_*N*_) is the joint entropy of the set.

Using *TC*, we then obtained coordination information (*CI*) which quantifies the amount of information shared by a set of variables of arbitrary size that is also shared with another variable:

8 $$CI(\{{X}_{1},\ldots ,{X}_{N}\}|{X}_{m})=TC({X}_{1},\ldots ,{X}_{N})-TC({X}_{1},\ldots ,{X}_{N}|{X}_{m})$$

In the above, $TC({X}_{1},\ldots ,{X}_{N}|{X}_{m})$ represents conditional total correlation between $\{{X}_{1},\ldots ,{X}_{N}\}$, conditioning on *X*_*m*_.

From *CI*, we calculated the mutual coordination information (MCI), defined as the amount of coordination information that is shared between two residues and the same set:

9 $$\begin{array}{c}MCI(\{{X}_{1},\ldots ,{X}_{N}\}|{X}_{m},{X}_{n})=CI(\{{X}_{1},\ldots ,{X}_{N}\}|{X}_{m})+CI(\{{X}_{1},\ldots ,{X}_{N}\}|{X}_{n})\\ -CI(\{{X}_{1},\ldots ,{X}_{N}\}|{X}_{m},{X}_{n})\end{array}$$

Here we have applied NbIT to all the non-hydrogen atoms of the mTMEM16F protein. The distal Ca^2+^ binding site (termed S1 site consisting of residues E395, S854, I857, and D859; and denoted E395/S854/I857/D859) was defined as the *transmitter*.

The selection of the *receiver* regions^42^ of the protein involved in allosteric coupling with the distal Ca^2+^ binding site during the groove opening process is a two-stage procedure. First, a set of NbIT sites was created, which included all the residue pairs along the TM2–TM4 and TM4–TM6 interfaces that form stable interactions prior to the groove opening. An interaction was considered stable if its occupancy was >75%; the cut-off for the minimum distance between the two sidechains was set to 3.5 Å for polar interactions, and to 6 Å for nonpolar ones. For this preliminary extended set, we evaluated the Coordination Information (*CI*) shared between each of these sites and the transmitter Ca^2+^ binding site, for all the microstates visited by the trajectories capturing the scrambling process. Since the highest values of *CI* were observed for microstate 8 (see “Results”), we focused on the *CI* values obtained for this microstate for further analysis of the most prominent receivers of the allosteric signal from the Ca^2+^ binding site. Specifically, the *CI* data obtained for microstate 8 was clustered with a Fisher-Jenks algorithm (https://github.com/mthh/jenkspy) into three groups of sites with various levels of allosteric coordination of the Ca^2+^ binding sites: low (*CI* < 24%), average (24% ≤ *CI* ≤ 50%), and high (*CI* > 50%). Residue pairs from each of the two interfaces that exhibited high levels of *CI* were then selected as the most prominent receiver sites in these regions. These sites were the following: S2 site (W359/Y502); S3 site (Q506/Q351); S4 site (Q351/Y502); S5 site (Y502/Q506); S6 site (M522/W619); S7 site (F518/K616); S8 site (S514/ Q608); S9 site (S514/F518/M522); S10 site (Q608/K616/W619). The allosteric channels connecting the distal Ca^2+^ binding site with the TM4–TM2 and TM4–TM6 interfaces were then identified by evaluating the MCI^42^ between the S1 site and the combined sites S2+S3+S4+S5, and S6+S7+S8.

### Statistics and reproducibility

The MSM analysis was performed on 544 replicates generated by the adaptive ensemble MD simulation protocol, as described above.

### Reporting summary

Further information on research design is available in the Nature Research Reporting Summary linked to this article.

## Supplementary information

Supplementary Information

Description of Additional Supplementary Files

Supplementary Data

Supplementary Movie 1

Supplementary Movie 2

Reporting Summary

## Data Availability

The MD simulation trajectories described in this work are available upon reasonable request. The source files used to visualize the tICA space in Fig. 3 are available as Supplementary Data 1–3.

## Code availability

The NbIT and the TCF codes used to perform quantitative allostery analyses described above are publicly available at the following github repositories: https://github.com/weinsteinlab/NbIT and https://github.com/weinsteinlab/TCF, respectively.
