# Supplementary material for: The allosteric mechanism leading to an open-groove lipid conductive state of the TMEM16F scramblase
Source: Commun Biol. 2022 Sep 19;5:990. doi: 10.1038/s42003-022-03930-8 (PMC9484709; doi:10.1038/s42003-022-03930-8)
Supplement: Supplementary file 1 — Supplementary Information [file 42003_2022_3930_MOESM1_ESM.pdf]

SUPPLEMENTAL INFORMATION

The allosteric mechanism leading to an open-groove lipid conductive state of the TMEM16F scramblase

George Khelashvili[1,2*], Ekaterina Kots[1], Xiaolu Cheng[1], Michael V Levine[1,2], Harel Weinstein[1,2]

[1] Department of Physiology and Biophysics, Weill Cornell Medicine, New York, NY, 10065, USA

[2] Institute for Computational Biomedicine, Weill Cornell Medicine, New York, NY 10065, USA

[*]**Corresponding Author**:

Address: Department of Physiology and Biophysics,

Weill Cornell Medicine,

1300 York Avenue, room LC-501C, New York, NY, 10065

E-mail: gek2009@med.cornell.edu

Phone: 212-746-6348

Fax: 212-746-6226

SUPPLEMENTAL FIGURES

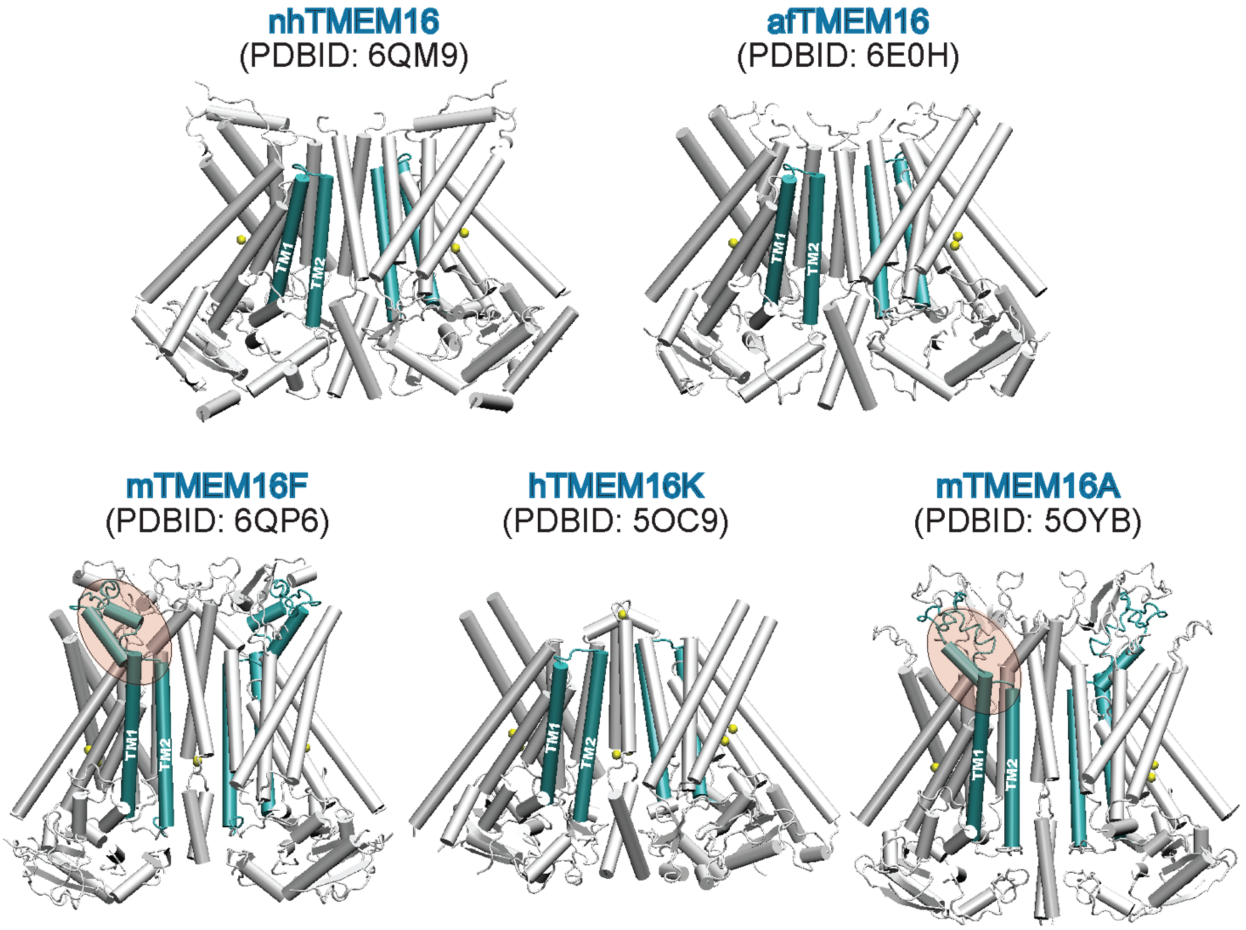

Figure S1: Differences in the characteristics of various structurally determined TMEM16 proteins. Structural comparison of nhTMEM16 (PDBID 6QM9), afTMEM16 (PDBID 6E0H), mTMEM16F (PDBID 6QP6), hTMEM16K (PDBID 5OC9), and mTMEM16A (PDBID 5OYB). TM1-TM2 helices and the connecting loop region are shown in cyan color on each structure. Note that in the mTMEM16F and mTMEM16A models the linker region connecting TMs 1 and 2 reaches towards the groove region (see also Figure 1 in the main text).

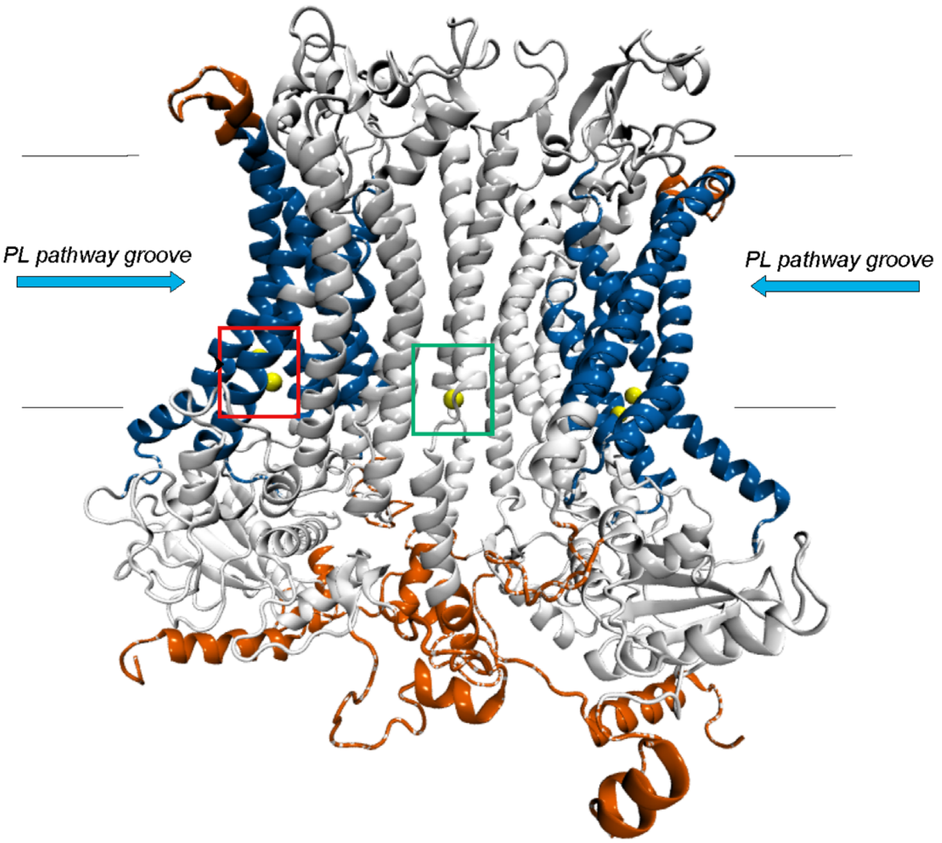

Figure S2: Structural model of the mTMEM16 phospholipid scramblase. The fragments missing from the cryo-EM structure (PDBID 6QP6), shown in red, were modeled as described in Methods. The phospholipid (PL) pathway (groove region) is rendered in blue in each protomer. The green box indicates the position of the 3rd, distal Ca^{2+} binding site at the dimer interface (all Ca^{2+} ions are shown as yellow spheres). Horizontal black lines demarcate the relative positions of upper and lower leaflets of the membrane (i.e., idealized positions of phosphate groups on the two leaflets).

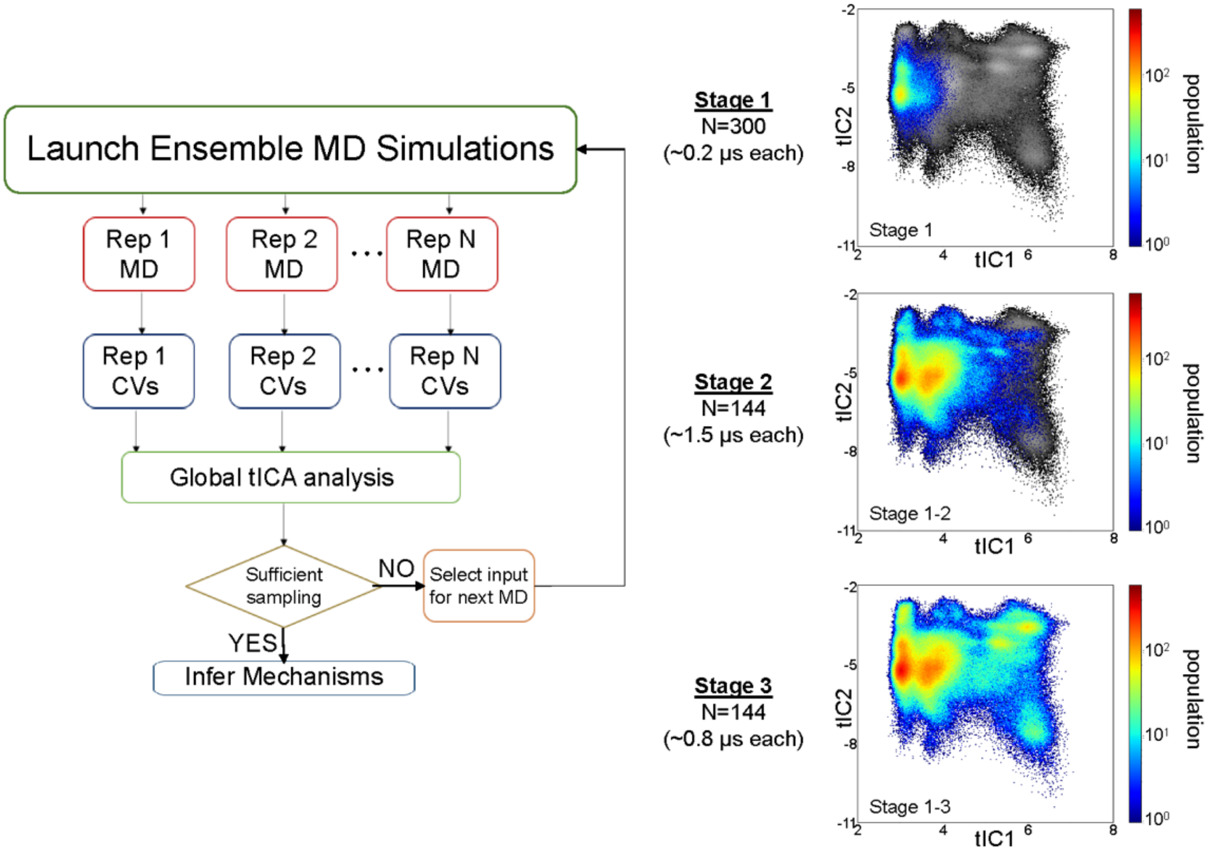

Figure S3: Protocol of the multi-stage adaptive ensemble MD simulation. ***Left panel:*** Flowchart detailing steps of the protocol. Each stage of the adaptive ensemble MD simulations consists of 1) running *N* number of independent replicates of the system; 2) Calculating collective variables (CVs) from each trajectory; and 3) Combining the CVs into a global tICA dimensionality reduction analysis. In each row, Rep 1 to Rep N represent the individual replicas run separately. If the sampling of the phase space is satisfactory, subsequent analyses are performed to infer kinetic and molecular mechanisms. If more sampling is required, a next round of ensemble MD simulations is launched from selected frames from the previous stage (see Methods for more details). Here, this adaptive protocol was carried out in 3 stages. The number of independent replicates for each state and lengths of MD simulation runs for each replicate in each stage are shown to the right of the Flowchart. ***Panels on the right:*** Projections of the frames from the trajectories obtained at each stage onto the final (Stage 3) 2D tICA space spanned by the first 2 tIC vectors – tIC1 and tIC2 (see Methods for details). The populations of the different states on the final tICA space are indicated by the colored portions in each stage, superimposed on a greyscale representation of the Stage 3 tICA space. Together, the three panels show the gradual increase of the tICA space sampling as the adaptive protocol is executed in 3 stages.

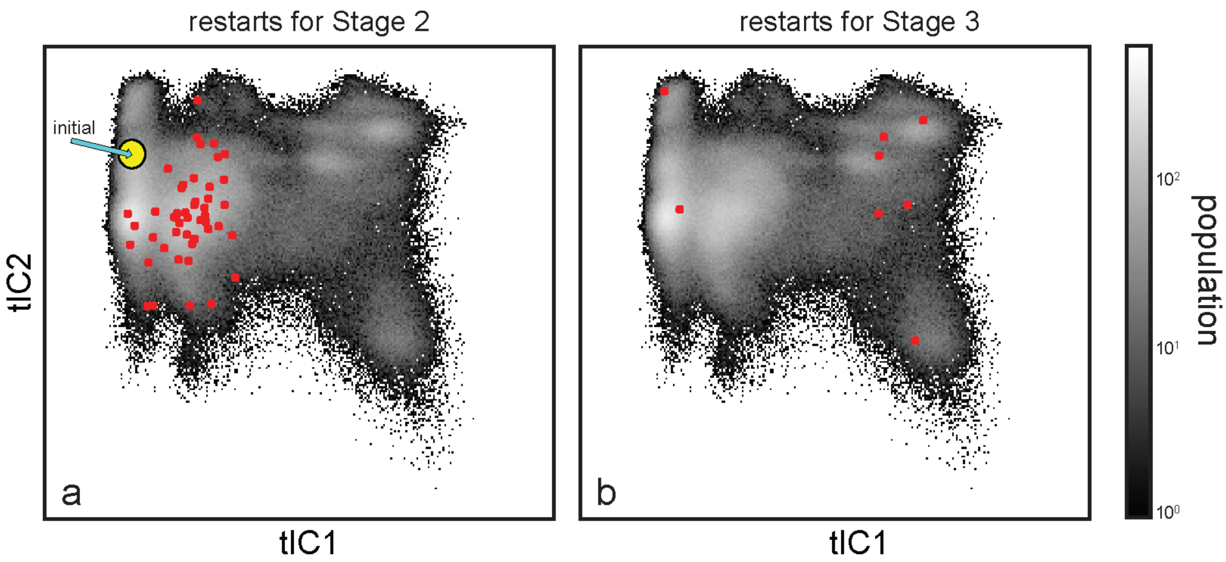

Figure S4: Restart frames of trajectories started in each stage of the adaptive ensemble MD simulation protocol. The restart frames are shown as red circles projected onto the final 2D tICA landscape shown in grayscale, for (**a**) Stage 1 and (**b**) Stage 2 simulations (see also Figure S3). The location on the tICA space of the initial, cryo-EM structure is also indicated in panel a by the yellow circle.

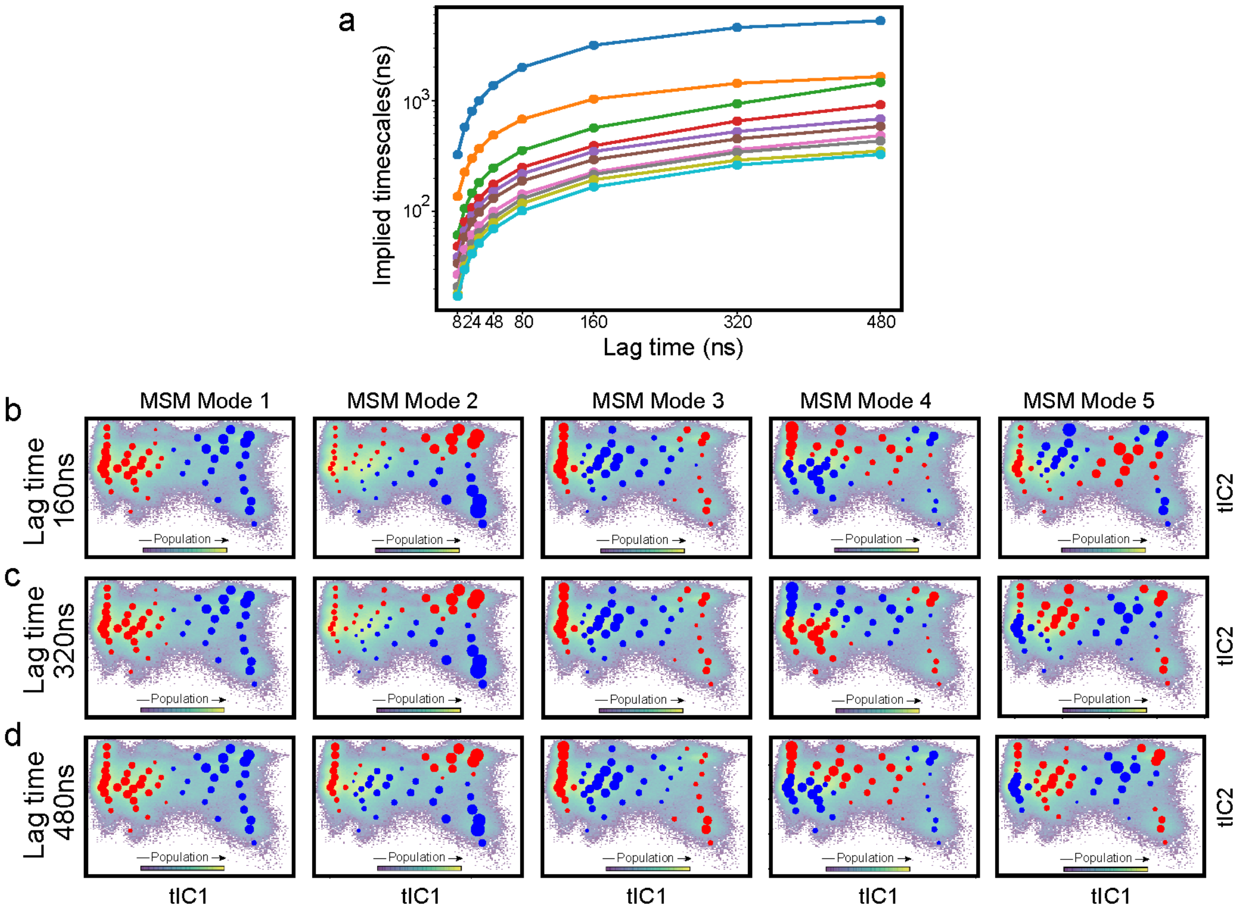

Figure S5: Choosing the lag time for the MSM analysis. (**a**) The implied timescales as a function of the lag time for the top 10 MSM relaxation modes (in different color). (**b-d**) The population exchanges between the microstates on the 2D tICA space with positive and negative eigenvectors along the top 5 MSM relaxation modes for lag times of 160 ns (b), 320 ns (c), and 480 ns (d). Red and blue circles on the tICA landscapes show the locations of the microstates with positive and negative contributions, respectively, to the relaxation modes. Microstates with larger circles contribute more to the relaxation mode (i.e., correspond to higher absolute value of the MSM eigenvector component). The color map of the tICA space in Panels b-d identifies the populations distribution of the different states of mTMEM16F with lighter and darker shades corresponding to the high and low density states, respectively.

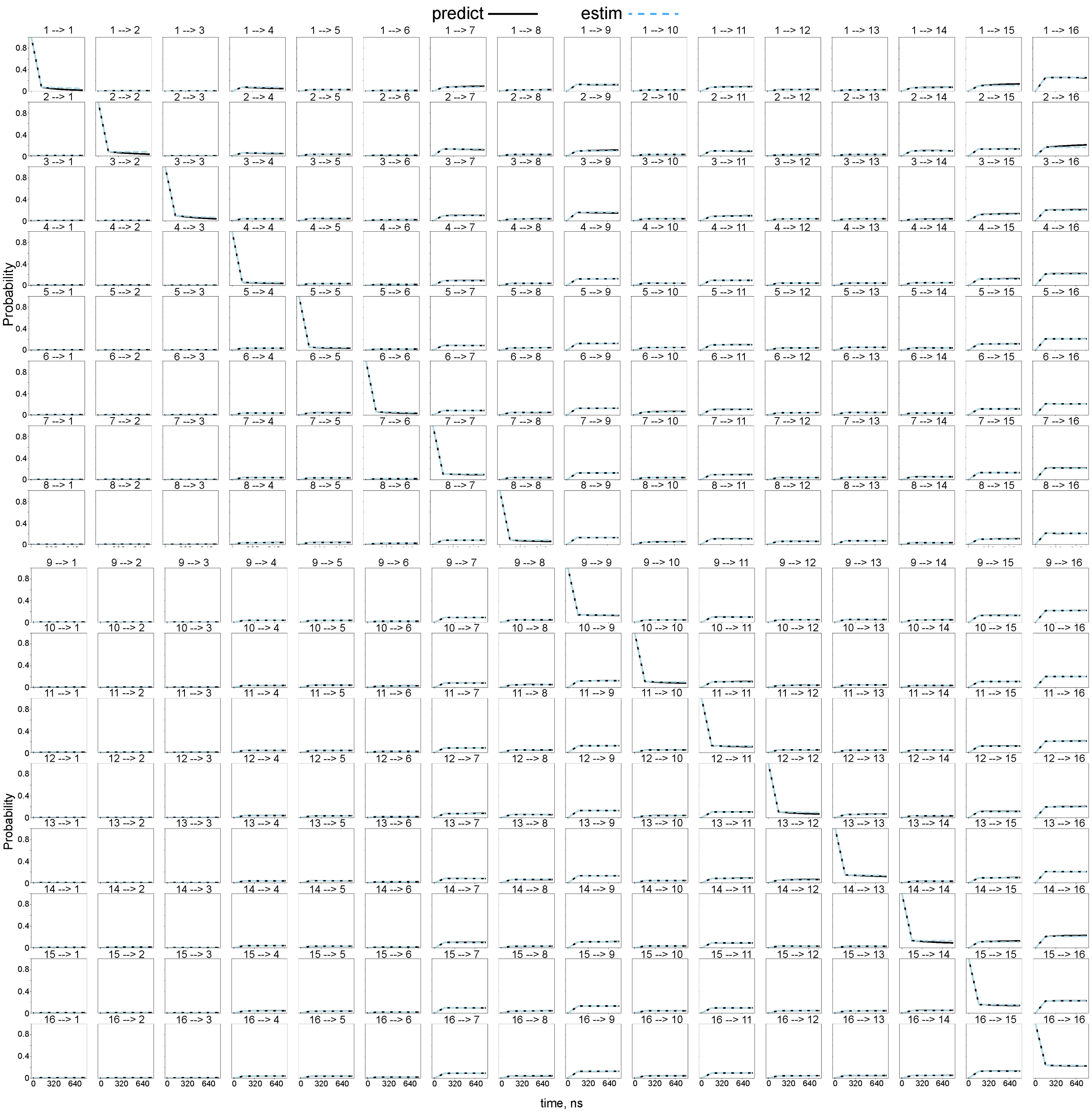

Figure S6: The Chapman-Kolmogorov test of the MSM. The test was performed on the MSM generated at 160ns lag time and using 16 kinetic macrostates. The probabilities for transitioning between all the indicated pairs of states predicted from the MSMs (solid lines) coincides with those estimated directly from the MD simulations (dotted lines).

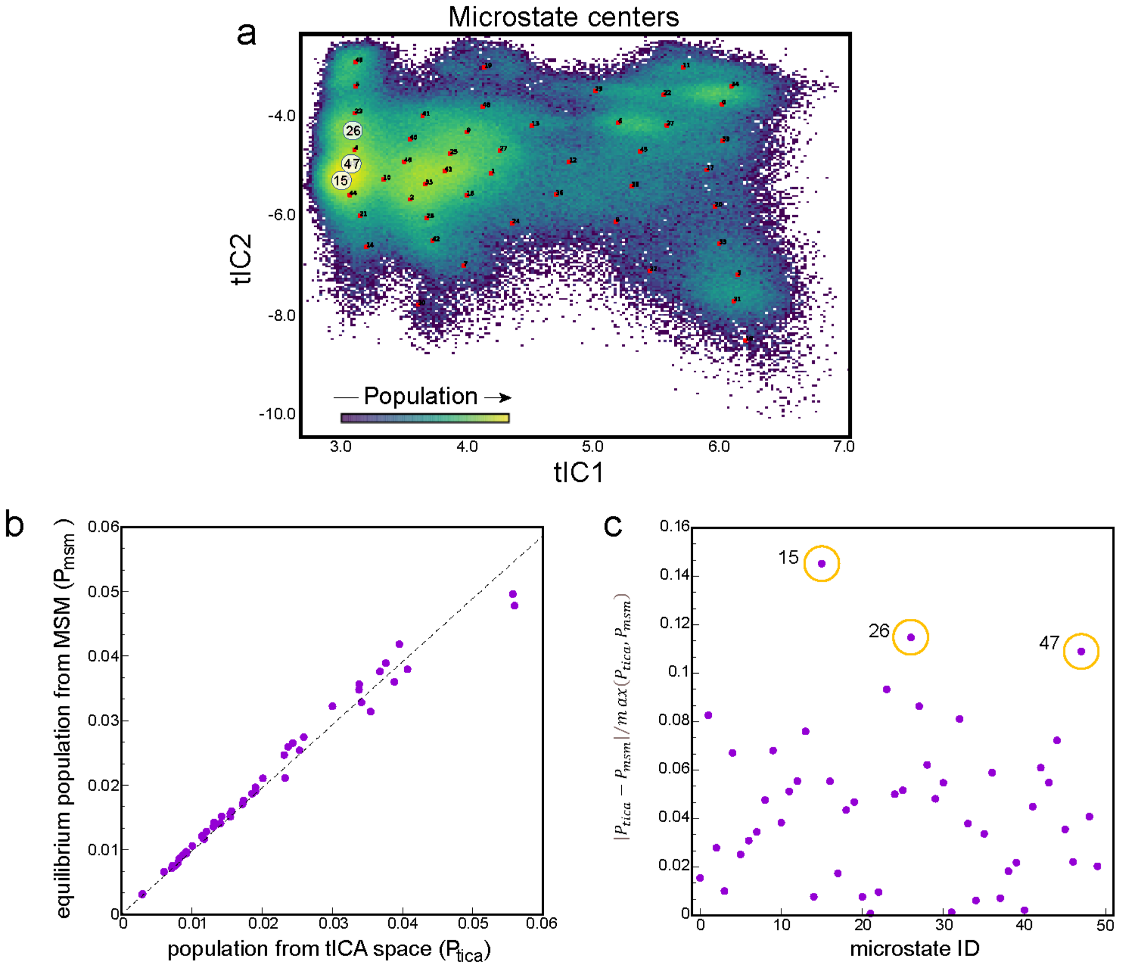

Figure S7: Populations of microstates sampled during the adaptive MD simulations are consistent with the equilibrium population distributions. (**a**) The 2D tICA space of the first two tIC vectors discretized in 50 microstates (red symbols represent the locations of the centers of the microstates). The color map of the tICA space identifies the populations distribution of the different states of mTMEM16F with lighter and darker shades corresponding to the high and low density states, respectively. (**b**) The microstate populations obtained from the tICA analysis (P_{tica}) are plotted against the equilibrium populations of the microstates from the MSMs (P_{msm}). The dotted line represents a linear fit to the data in the form of the function f(x)=0.98x (**c**) The absolute value of the difference between the two populations from panel B normalized by the largest population ($|P_{tica} - P_{msm}|/\max(P_{tica}, P_{msm})$) are plotted for each microstate. For the majority of the microstates the difference between the two populations is < 10%. The 3 microstates (15, 26, and 47) for which the difference is above 10% correspond to the phase space region from Stage 1 simulations where the microstates are highly populated as expected.

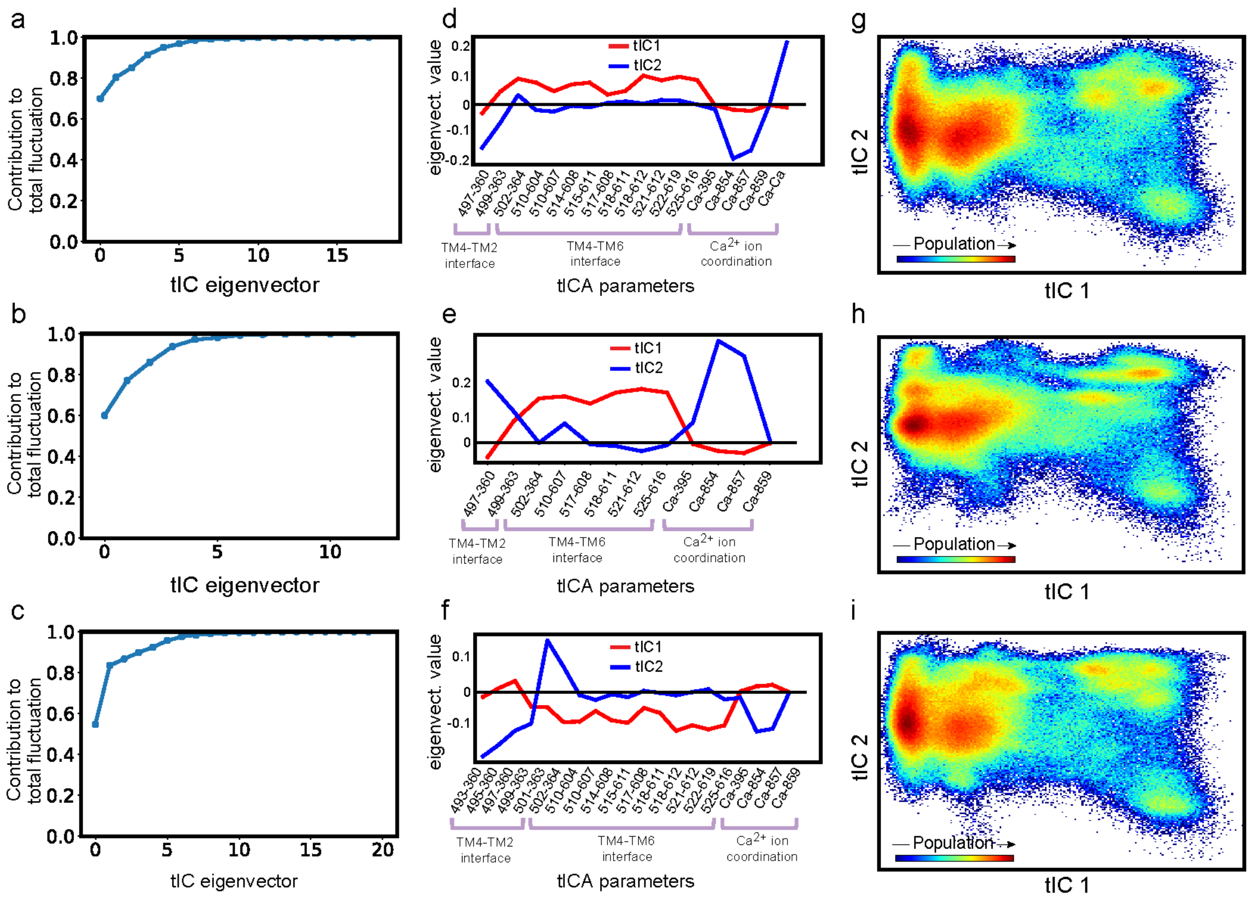

Figure S8: tICA analysis using modified CV definitions. Panels (**a-c**) show the contributions of tIC eigenvectors to total fluctuations; (**d-f**) show contributions of the CVs used as tICA parameters to the tIC1 and tIC2 vectors; panels (**g-i**) show the 2D tIC landscapes for the 3 corresponding sets of CVs. These modified CVs differ from the originally selected parameters (presented in Fig. 2a) in the following ways: (i)-addition of a CV describing the distance between the two Ca2+ ions bound in the distal sites of the two monomers of mTMEM16F (panels a, d, g); (ii)-removal of 5 out of 11 CVs describing the dynamics of TM4-TM6 interface (panels b, e, h); and (iii)-addition of 3 CVs describing the dynamics of the TM4-TM2 interface by the pairwise residue distances: 493-360, 495-360, and 501-360 (panels c, f, i). The color map of the tICA space in Panels g-i identifies the populations distribution of the different states of mTMEM16F as in Figure 3.

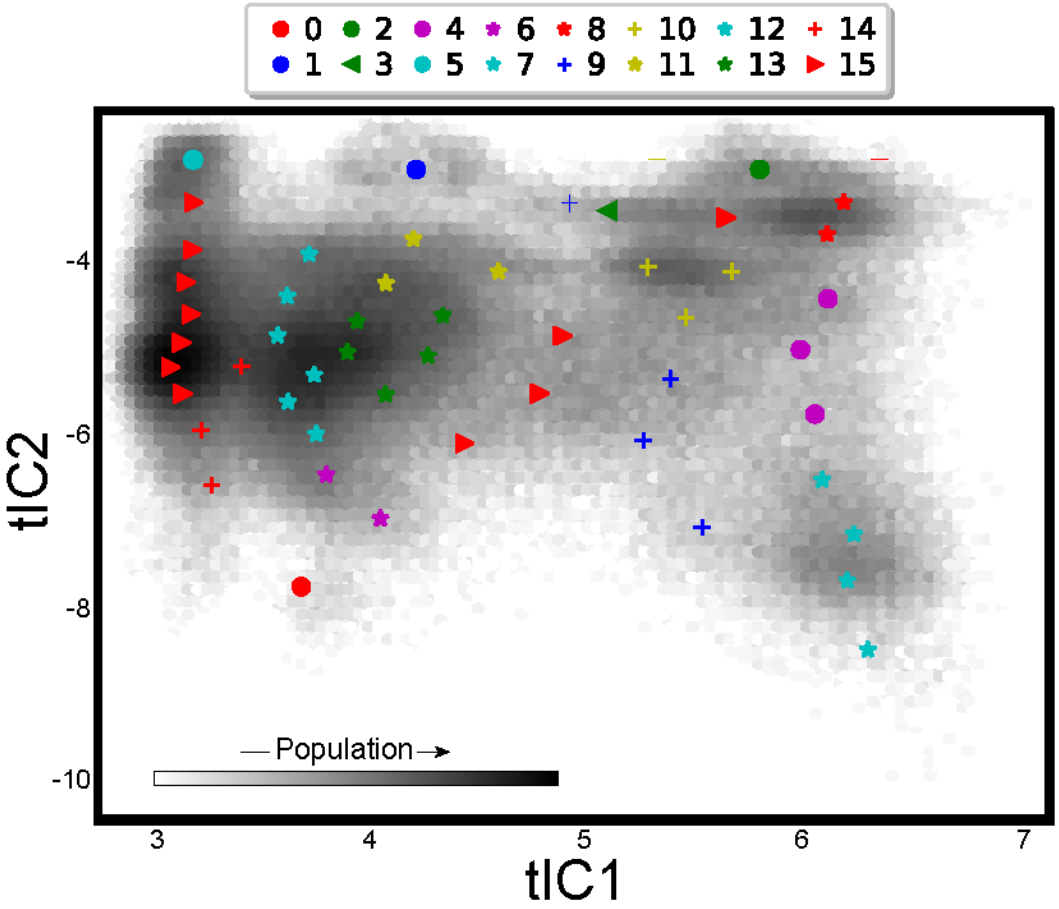

Figure S9: Discretization of the 2D space of the first two tIC vectors into structurally similar microstates and kinetically similar macrostates. The 2D space of tIC1 and tIC2 vectors (in grey) is discretized into 50 microstates. The centroids of these structurally similar microstates are shown as symbols of various colors and sizes, according to their grouping into 16 macrostates based on kinetic similarity (see Methods). The color map of the tICA space identifies the populations distribution of the different states of mTMEM16F with darker and lighter shades corresponding to the high and low density states, respectively.

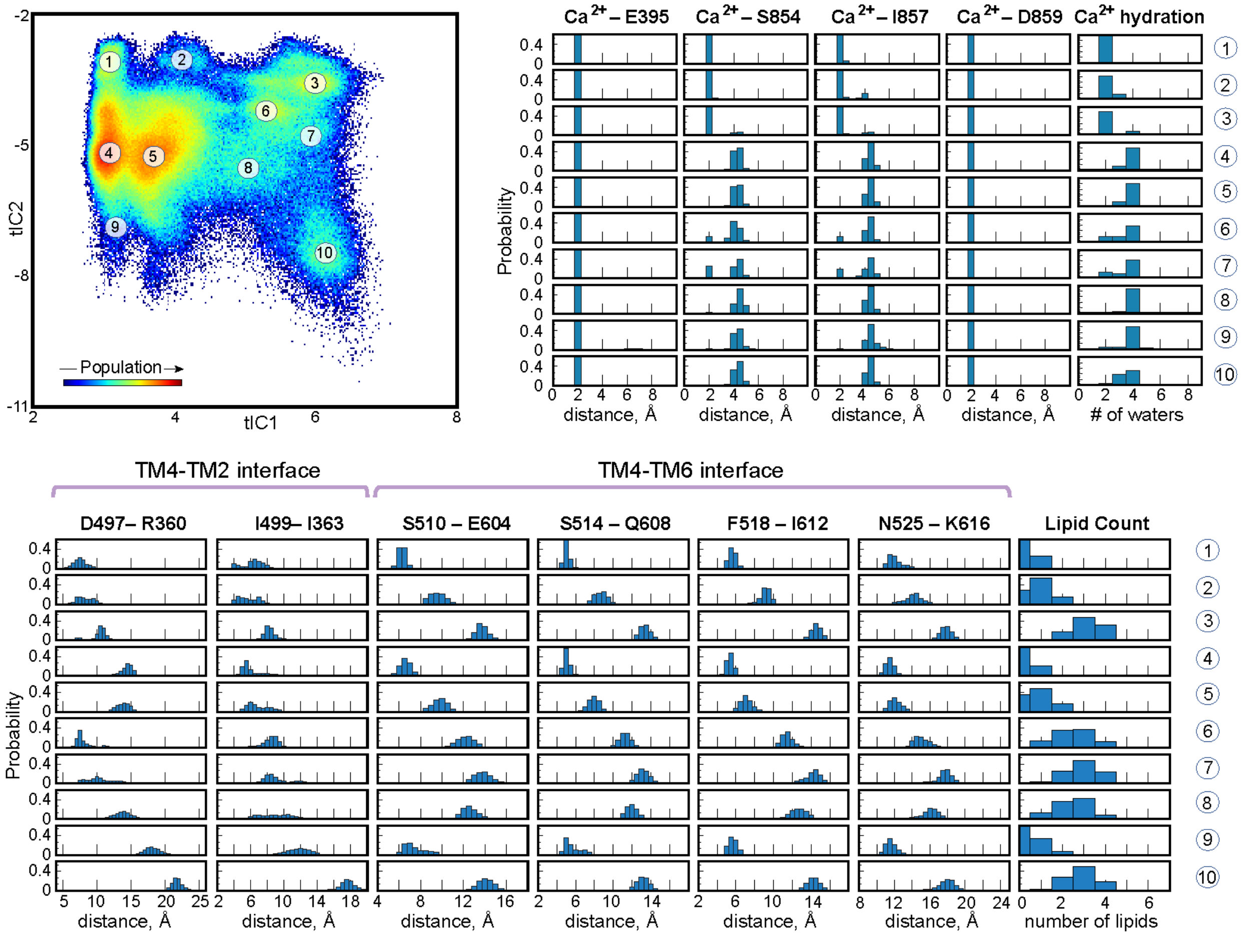

Figure S10: Structural characteristics of various microstates on the tICA space. The positions of selected microstates are indicated on the 2D space of the first two tIC vectors (as in Figure 3). The structural characteristics of the TM4-TM6 interface, TM4-TM2 interface, and the Ca^{2+} ion binding site are quantified in the accompanying histograms for each of the selected microstates. The # of waters entry is defined as the number of oxygen atoms at a distance of 3Å from the distal Ca^{2+} ion. The number of lipids entry is defined as the number of lipid phosphorus atoms within 7Å of residues 467, 471, 475, 514, 517, 521, 559, 563, 566, 570, 574, 612, 608, and 604.

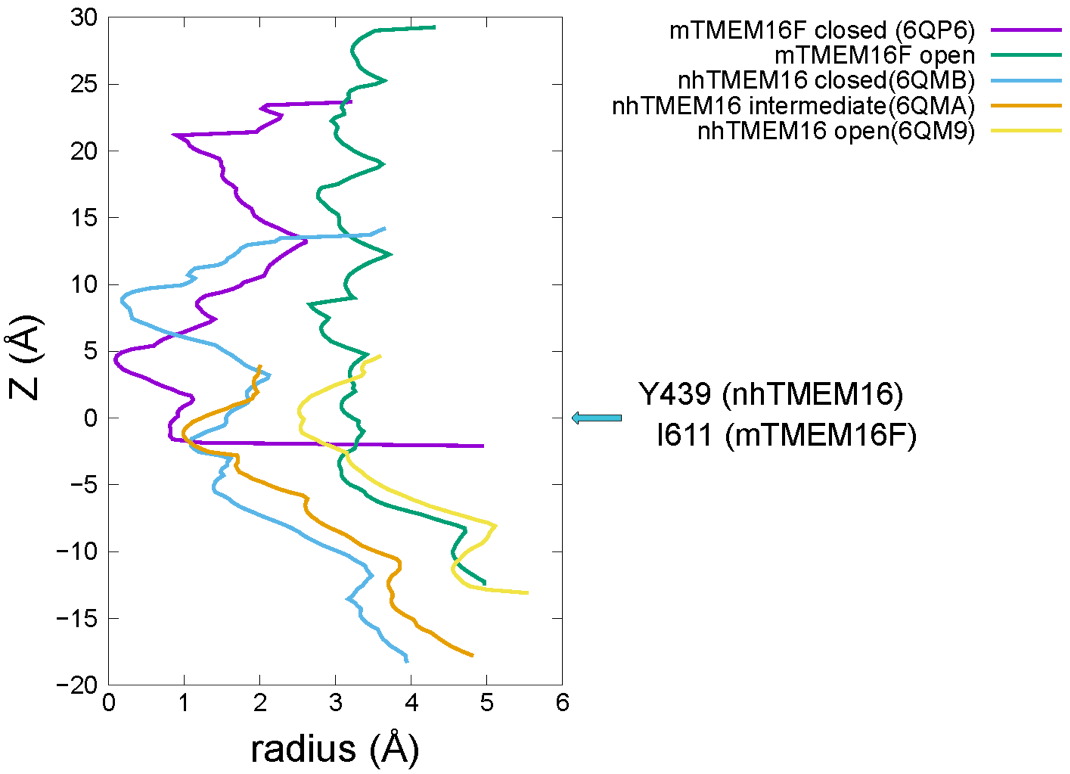

Figure S11: Comparison of pore dimensions in the open groove conformation of mTMEM16F to the open groove structure of nhTMEM16. The radius of the permeation pathway in nhTMEM16 and mTMEM16F was estimated using the program HOLE (http://www.holeprogram.org/) for the various states, as indicated. Purple: Closed groove structure of mTMEM16F (PDBID 6QP6); Green: Open groove structure of mTMEM16F (representative snapshot from microstate 10 in Figure 3); Blue: Closed groove structure of nhTMEM16 Open (PDBID 6QMB); Orange: Intermediate groove structure in nhTMEM16 (PDBID 6QMA); Yellow: Open groove structure of nhTMEM16 (PDBID 6QM9). The cyan-color arrow marks the Z=0Å coordinate and indicates the position of the reference residue in nhTMEM16 (Y439), and in mTMEM16F (I611), along the z axis.

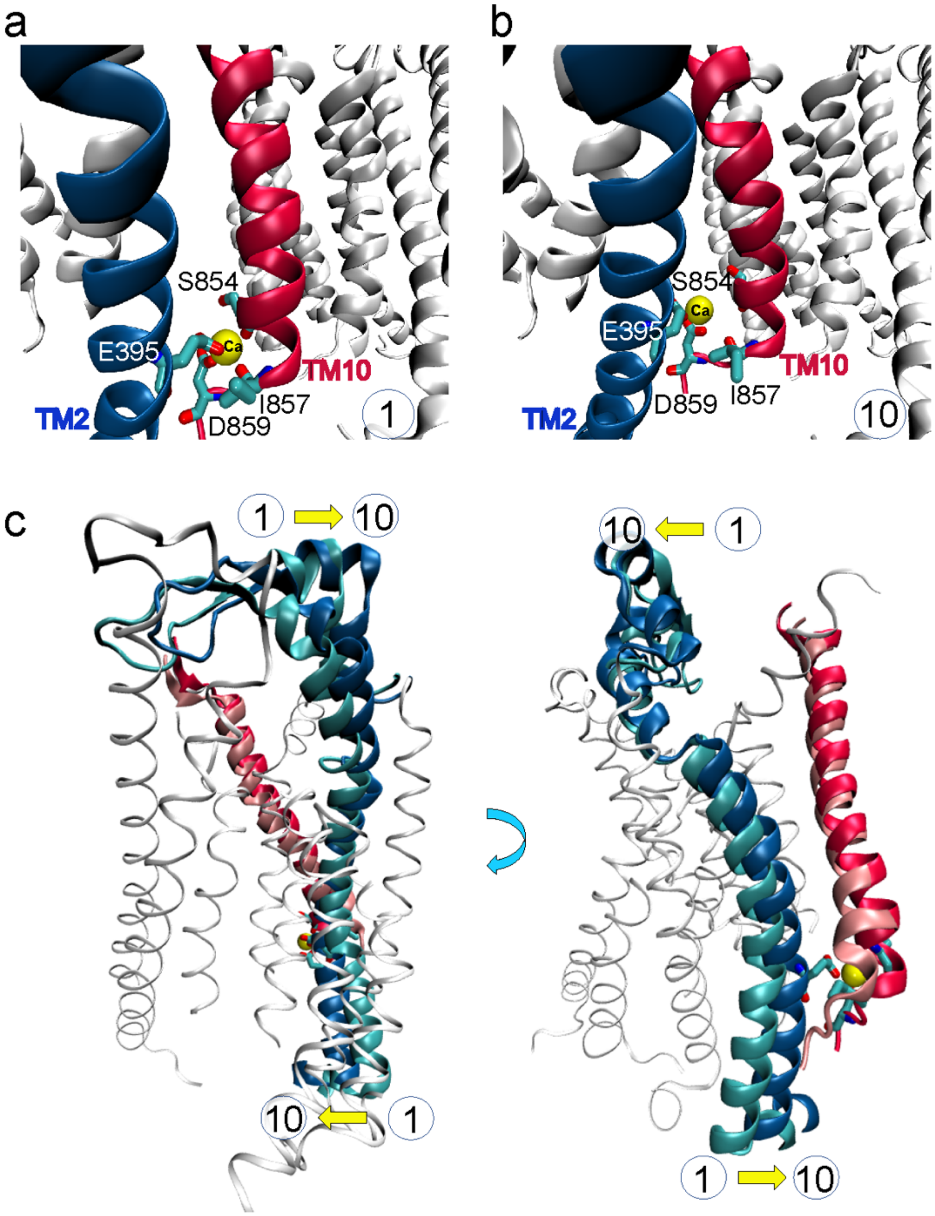

Figure S12: Conformational changes in the distal Ca^{2+} ion binding site at the mTMEM16F dimer interface. **(a)** The Ca^{2+} ion (yellow sphere) is stably bound in the distal binding site, coordinated by the sidechains of E395 and D859, as well as by the backbone carbonyls of S854 and I857. This interaction mode is representative of *microstate 1* in Figure 3. **(b)** Ca^{2+} ion is destabilized in the binding site. It has lost contact with the backbone carbonyls and is now coordinated solely by the sidechains of E395 and D859 residues. This interaction mode is representative of *microstate 10* in Figure 3. TM2 and TM10 helices where the coordinating residues reside are shown in blue and red color, respectively. **(c)** Two views of the mTMEM16F monomer highlighting conformational changes in TM2 and TM10 during the transition from *microstate 1 to 10*. The conformations of TM2 in microstates 1 and 10 are shown in cyan and blue color, respectively, whereas conformations of TM10 helix in microstates 1 and 10 are depicted in pink and red, respectively. The conformation of the rest of the protein structure is from microstate 10.

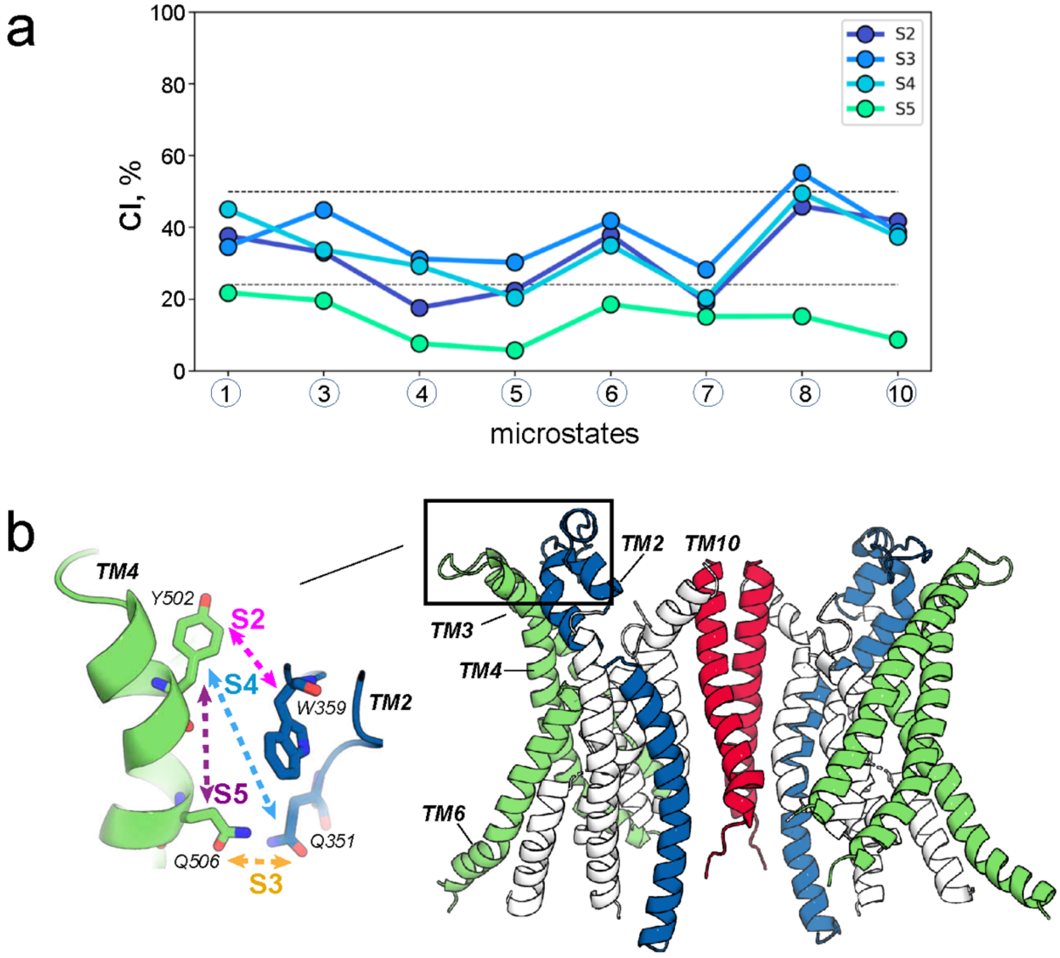

Figure S13: Allosteric coupling between the distal Ca^{2+} binding site and the TM4-TM2 interface of the groove region in mTMEM16F. (**a**) The coordination information (***CI***) between site S1 (non-hydrogen atoms of the four Ca^{2+} coordinating residues: D395, S854, I857, D859) and several sites at the TM4-TM2 interface (S2 site – non-hydrogen atoms of Y502-W359 pair of residues; S3 site – non-hydrogen atoms of Q504-Q351 pair of residues; S4 site – non-hydrogen atoms of Y502-Q351 pair of residues; S5 site – non-hydrogen atoms of Y502-Q506 pair of residues) were calculated from the trajectories representing the indicated microstates (see also Figure 5). The horizontal lines demarcate regions of *low*, *average*, and *high* levels of coordination as obtained from the clustering of the *CI* data using the Fisher-Jenks algorithm (see Methods). (**b**) The mTMEM16F dimer structure highlighting the location and composition of the S2, S3, S4, and S5 sites used in the calculations of *CI* shown in panel a.

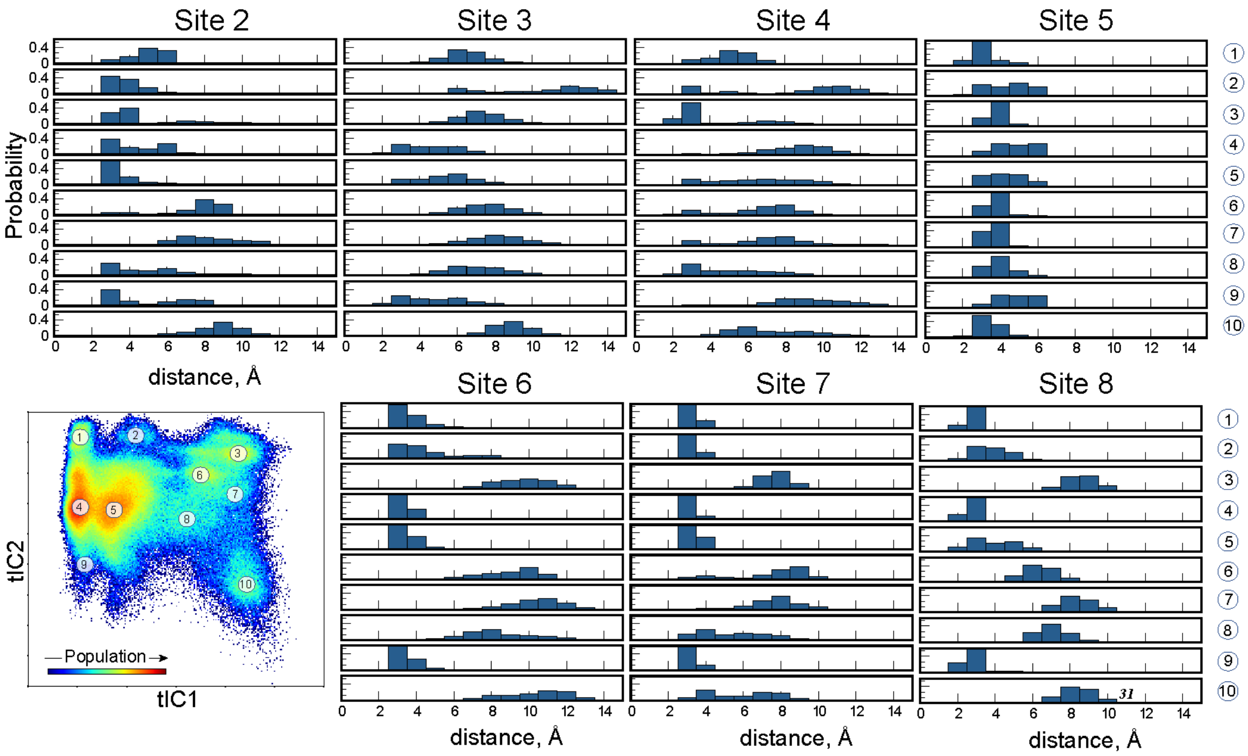

Figure S14: Structural characteristics of the various NbIT sites. Pair-wise minimum distances of the residues at NbIT *receiver* Sites 2-8 in selected microstates of the 2D tICA space. S2 site – W359/Y502; S3 site – Q506/Q351; S4 site – Q351/Y502; S5 site – Y502/Q506; S6 site – M522/W619; S7 site – F518/K616; S8 site – S514/ Q608; S9 site – S514/F518/M522; S10 site – Q608/K616/W619. The color map of the tICA space identifies the populations distribution of the different states of mTMEM16F as in Figure 3.

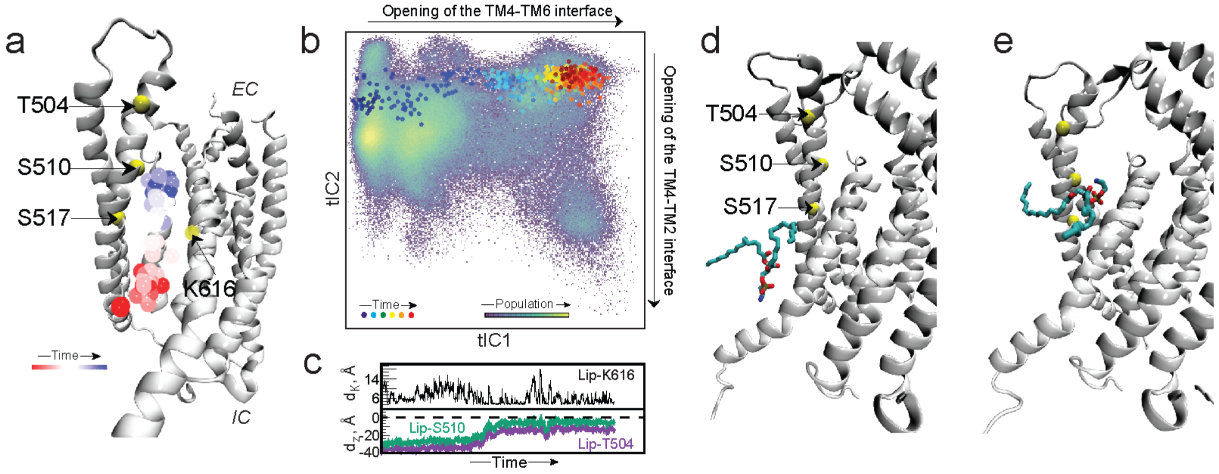

Figure S15: Incomplete lipid scrambling event in the MD simulations. (**a**) The protein monomer is shown in cartoon, in the the structure taken from the final frame of the respective simulation trajectory, and the trajectory of the phosphorus atom of the translocated lipid (from the IC to EC side) is shown as spheres colored according to the timestep (see "Time" color bar). The C_α atoms of residues T504, S510, and S517 are shown as yellow spheres. (**b**) The time evolution of the corresponding MD trajectory projected onto the 2D tICA landscape from Figure 3 is shown by large colored dots. The darker colors (blue, cyan) indicate the initial stages of the simulation, lighter colored dots (yellow, green) indicate the middle part of the trajectory, and red shades show the last third of the trajectory. The vertical arrow on the right indicates the direction along which the TM4-TM6 and TM4-TM2 interfaces open on the tICA space. The color map of the tICA space identifies the populations distribution of the different states of mTMEM16F with lighter and darker shades corresponding to the high and low density states, respectively. (**c**) The time-evolution of the distance between the phosphorus atom of the scrambled lipid and the amine nitrogen atom of residue K616 sidechain is shown (d_K, black traces), and the time-evolution of the Z-distance between the headgroup of the translocated lipid and the C_α atom of residues T504 and S510 (d_Z^{S504} and d_Z^{S510}) is shown by the purple and green traces, respectively. (**d-e**) Snapshots from the first (**d**) and the last (**e**) frames of the trajectory showing the positioning of the translocated lipid. The color code for the protein is the same as in panel **a**. Note that, while the lipid is geometrically flipped during the simulation, it is still bound in the groove as shown by the d_Z^{S517} distance remaining < 0 (see panel **b**). The headgroups of POPE and POPG lipids were defined as the center-of-mass of the following group of atoms (in CHARMM36 nomenclature): POPE (N, C12, C11, P); POPG (C13, OC3, C12, C11, P).

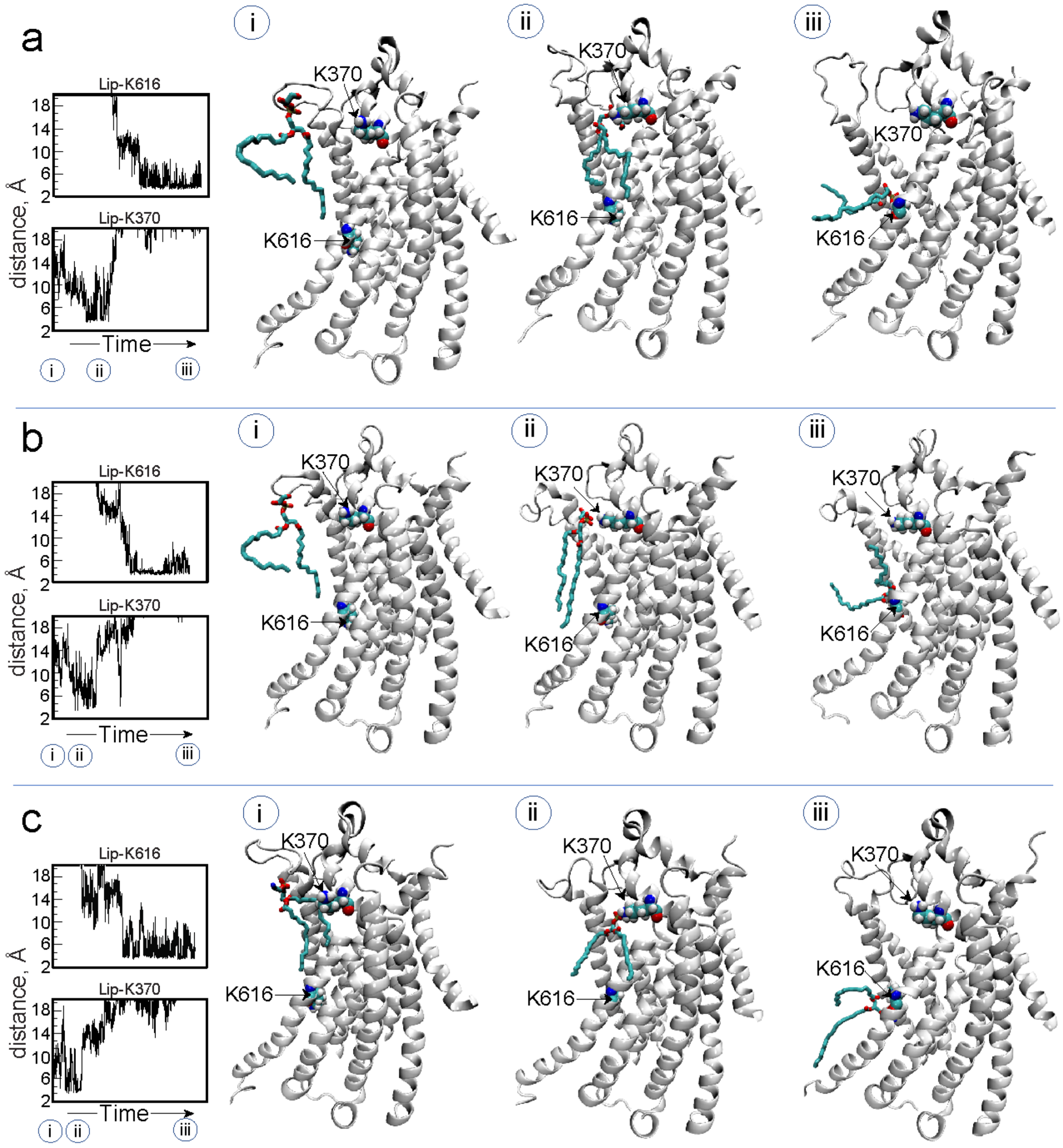

Figure S16: The scrambled lipid is coordinated by basic residues in the groove area. Panels **a, b,** and **c** show coordination by the positively charged residues of the lipid translocated from the EC to IC side of the groove in the three trajectories described in Figure 6 (panels d, b, and a, respectively). In each panel, the left graphs show the time-evolution of the distance between the phosphorus atom of the scrambled lipid and the nitrogen of the amine group of residues K616 and K370. The snapshots show the configuration of the system at three specified time points (marked as **i, ii,** and **iii**) along the respective trajectory. In these snapshots, the mTMEM16F monomer is drawn in white cartoon, the scrambled lipid is in licorice and two basic residues in the groove area, K616 and K370 are shown in space-fill representation.

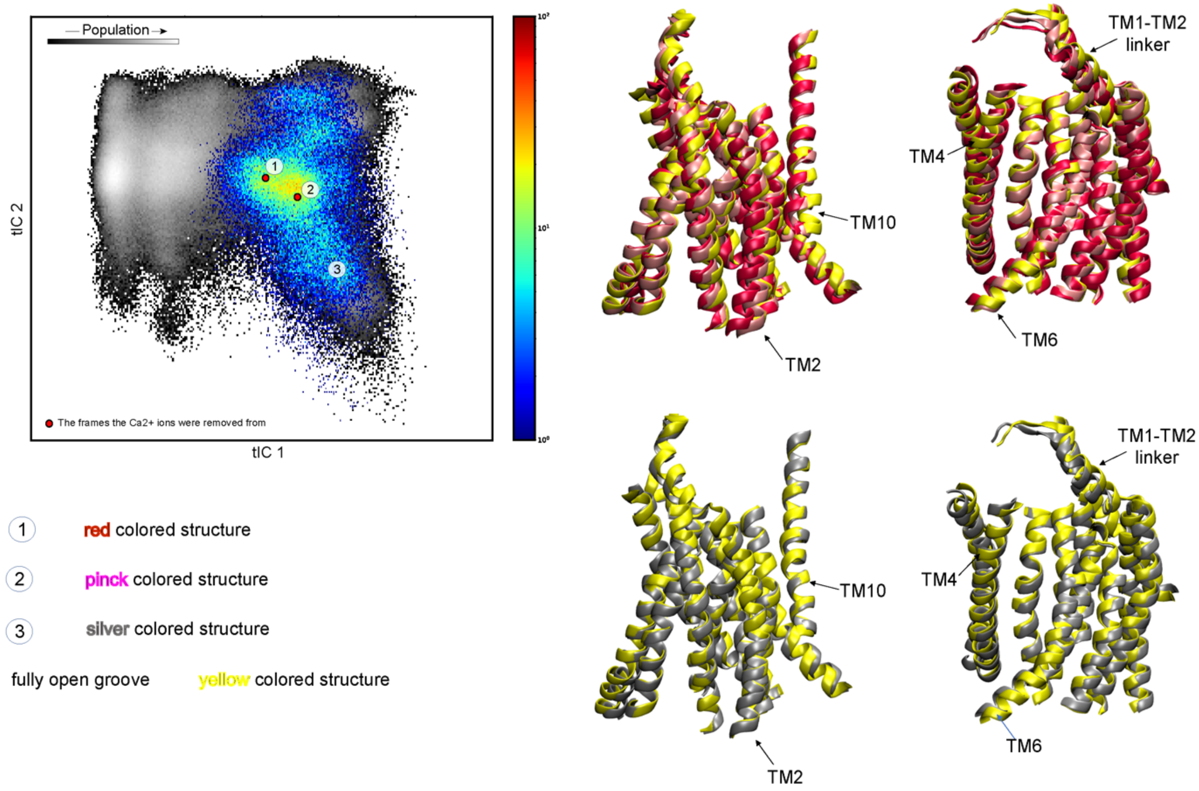

Figure S17: Conformational sampling of the mTMEM16F system with no Ca^{2+} ions bound in the distal site. Shown is the projection of the trajectory frames (color-coded points) onto the original 2D tICA space of the Ca^{2+} - bound protein system presented in Figure 3 of the main text (the color map of the tICA space identifies the populations distribution of the different states of mTMEM16F with lighter and darker shades corresponding to the high and low density states, respectively). The projections of the two trajectory frames in which Ca^{2+} ions were removed to initiate the new set of simulations are shown are large red circles. Note that, since no Ca^{2+} ions are bound in the distal site, to perform the tICA analysis, we replaced the CVs describing Ca^{2+} coordination (i.e., Ca^{2+} - protein residue distances) with the pairwise distances between the Ca^{2+} coordinating residues.

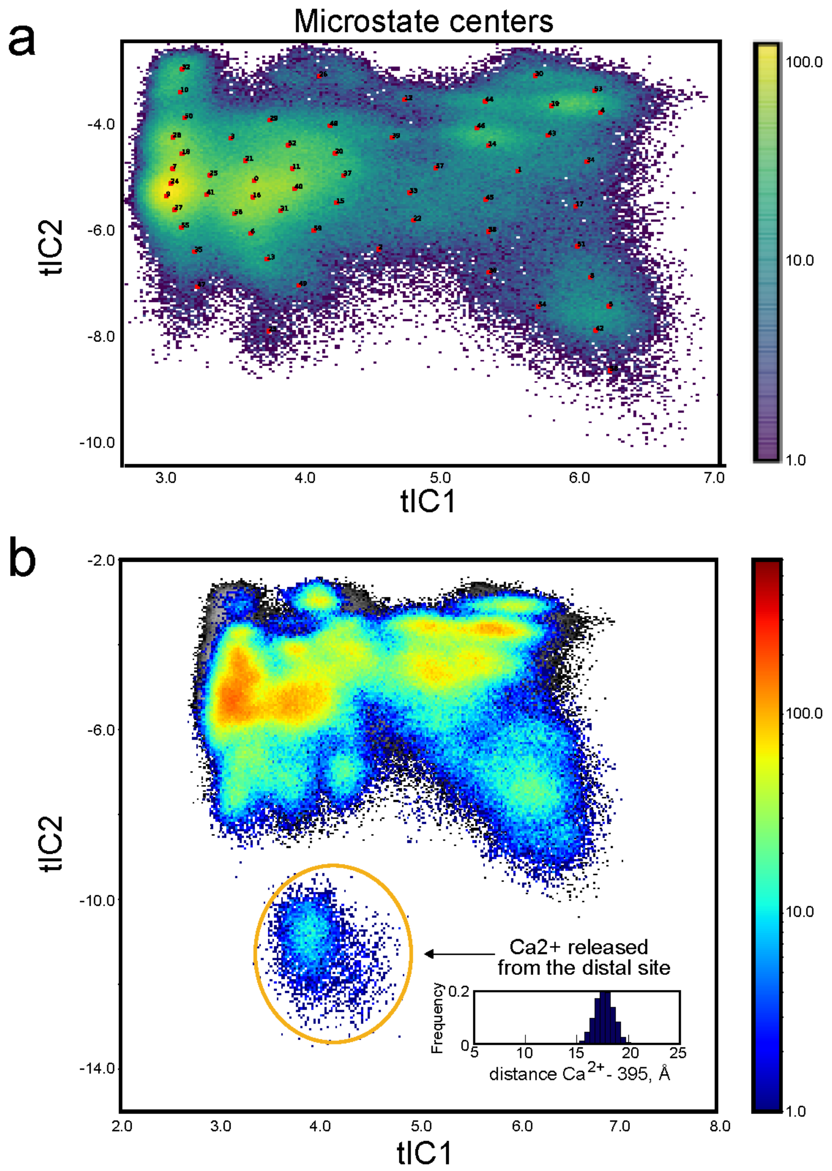

Figure S18: tICA landscape from the analysis of unbiased MD simulations. (**a**) Discretization of the 2D tICA space from Figure 3 of the main text into 60 microstates (the microstate centers are shown as red symbols and labeled). The color map of the tICA space identifies the populations distribution of the different states of mTMEM16F with lighter and darker shades corresponding to the high and low density states, respectively. (**b**) The projection of the trajectory frames from the unbiased MD simulations of the 60 microstate centers onto the tICA space from the top panel. The projected points are colored according to their population, and the original tICA space is shown as grey points. Note the encircled population of states which corresponds to the conformations with the Ca^{2+} ion released from its distal binding site (the accompanying histogram quantifies the distance between residue D395 in the binding site and the released Ca^{2+} ion. The position of this population along tIC2 axis corresponds to the open groove state (see Figure S17).
