# Supplementary material for: The allosteric mechanism leading to an open-groove lipid conductive state of the TMEM16F scramblase
Source: Commun Biol. 2022 Sep 19;5:990. doi: 10.1038/s42003-022-03930-8 (PMC9484709; doi:10.1038/s42003-022-03930-8)
Supplement: Supplementary file 2 — Description of Additional Supplementary Files [file 42003_2022_3930_MOESM2_ESM.pdf]

Description of Additional Supplementary Files

File name: Supplementary Data 1
Description: Source Data for Figure 3, tIC1

File name: Supplementary Data 2
Description: Source Data for Figure 3, tIC2

File name: Supplementary Data 3
Description: Source Data for Figure 3, tICA space.

File name: Supplementary Movie 1
Description: Molecular Dynamics trajectory showing lipid scrambling event from the intracellular to extracellular side.

File name: Supplementary Movie 2
Description: Molecular Dynamics trajectory showing lipid scrambling event from the extracellular to intracellular side.
