# Supplementary material for: The allosteric mechanism leading to an open-groove lipid conductive state of the TMEM16F scramblase
Source: Commun Biol. 2022 Sep 19;5:990. doi: 10.1038/s42003-022-03930-8 (PMC9484709; doi:10.1038/s42003-022-03930-8)
Supplement: Supplementary file 6 — Reporting Summary [file 42003_2022_3930_MOESM6_ESM.pdf]

nature research

Corresponding author(s): George Khelashvili

Last updated by author(s): 07/26/2022

Reporting Summary

Nature Research wishes to improve the reproducibility of the work that we publish. This form provides structure for consistency and transparency in reporting. For further information on Nature Research policies, see Authors & Referees and the Editorial Policy Checklist.

Statistics

For all statistical analyses, confirm that the following items are present in the figure legend, table legend, main text, or Methods section.

n/a	Confirmed	
☐	☒	The exact sample size (n) for each experimental group/condition, given as a discrete number and unit of measurement
☐	☒	A statement on whether measurements were taken from distinct samples or whether the same sample was measured repeatedly
☒	☐	The statistical test(s) used AND whether they are one- or two-sided<br>*Only common tests should be described solely by name; describe more complex techniques in the Methods section.*
☒	☐	A description of all covariates tested
☒	☐	A description of any assumptions or corrections, such as tests of normality and adjustment for multiple comparisons
☐	☒	A full description of the statistical parameters including central tendency (e.g. means) or other basic estimates (e.g. regression coefficient) AND variation (e.g. standard deviation) or associated estimates of uncertainty (e.g. confidence intervals)
☒	☐	For null hypothesis testing, the test statistic (e.g. F, t, r) with confidence intervals, effect sizes, degrees of freedom and P value noted<br>*Give P values as exact values whenever suitable.*
☒	☐	For Bayesian analysis, information on the choice of priors and Markov chain Monte Carlo settings
☒	☐	For hierarchical and complex designs, identification of the appropriate level for tests and full reporting of outcomes
☒	☐	Estimates of effect sizes (e.g. Cohen's d, Pearson's r), indicating how they were calculated

Our web collection on statistics for biologists contains articles on many of the points above.

Software and code

Policy information about availability of computer code

Data collection	For the molecular constructs used in computational experiments, we utilized Modeller version 9v1 and VMD version 1.9.1. To carry out atomistic molecular dynamics simulations, we used NAMD version 2.13 and OpenMM version 7.4.
Data analysis	Computational analysis was carried out using a combination of VMD version 1.9.1, MSM builder python scripts, and in-house scripts based on python (NbIT code) or R (TCF code) scripts. The NbIT and TCF codes, developed in our lab, have been publicly available at github as stated in the Code availability statement of the manuscript.

For manuscripts utilizing custom algorithms or software that are central to the research but not yet described in published literature, software must be made available to editors/reviewers. We strongly encourage code deposition in a community repository (e.g. GitHub). See the Nature Research guidelines for submitting code & software for further information.

Data

Policy information about availability of data

All manuscripts must include a data availability statement. This statement should provide the following information, where applicable:

- Accession codes, unique identifiers, or web links for publicly available datasets
- A list of figures that have associated raw data
- A description of any restrictions on data availability

Final masked and unmasked cryo-EM maps have been deposited in the EMDB database under the accession codes EMD-20221. The map with C1 symmetry which was used to compare the membrane environment and an unfiltered map that was used to aid model building were also included. Atomic coordinates have been deposited in the PDB database under the accession code 6OY3. The structure of the deposited PDB file is described in Figure 7.

Field-specific reporting

Please select the one below that is the best fit for your research. If you are not sure, read the appropriate sections before making your selection.

☒ Life sciences ☐ Behavioural & social sciences ☐ Ecological, evolutionary & environmental sciences

For a reference copy of the document with all sections, see nature.com/documents/nr-reporting-summary-flat.pdf

Life sciences study design

All studies must disclose on these points even when the disclosure is negative.

Sample size	MD simulations were carried out in 3 stages, each stage consisting of multiple replicates (stage 1 - 300; stage 2 - 144; stage 3 - 144).
Data exclusions	N/A
Replication	Data was replicated in multiple samples from independent simulations as stated above
Randomization	Data was not randomized
Blinding	Data was not blinded

Reporting for specific materials, systems and methods

We require information from authors about some types of materials, experimental systems and methods used in many studies. Here, indicate whether each material, system or method listed is relevant to your study. If you are not sure if a list item applies to your research, read the appropriate section before selecting a response.

Materials & experimental systems

n/a	Involved in the study	
☒	☐	Antibodies
☒	☐	Eukaryotic cell lines
☒	☐	Palaeontology
☒	☐	Animals and other organisms
☒	☐	Human research participants
☒	☐	Clinical data

Methods

n/a	Involved in the study	
☒	☐	ChIP-seq
☒	☐	Flow cytometry
☒	☐	MRI-based neuroimaging
